# Defective minor spliceosome mRNA processing results in isolated familial growth hormone deficiency

**DOI:** 10.1002/emmm.201303573

**Published:** 2014-01-30

**Authors:** Jesús Argente, Raquel Flores, Armand Gutiérrez-Arumí, Bhupendra Verma, Gabriel Á Martos-Moreno, Ivon Cuscó, Ali Oghabian, Julie A Chowen, Mikko J Frilander, Luis A Pérez-Jurado

**Affiliations:** 1 Departments of Endocrinology and Pediatrics, Hospital Infantil Universitario Niño Jesús, Universidad Autónoma de MadridMadrid, Spain; 2 Instituto de Investigación La PrincesaMadrid, Spain; 3 Centro de Investigación Biomédica en Red de Fisiopatología de la Obesidad y Nutrición (CIBEROBN)Madrid, Spain; 4 Genetics Unit, Universitat Pompeu FabraBarcelona, Spain; 5 Instituto de Investigación Hospital del Mar (IMIM)Barcelona, Spain; 6 Centro de Investigación Biomédica en Red de Enfermedades Raras (CIBERER)Barcelona, Spain; 7 Institute of Biotechnology, University of HelsinkiHelsinki, Finland

**Keywords:** mRNA splicing, pituitary hypoplasia, U12-type introns

## Abstract

The molecular basis of a significant number of cases of isolated growth hormone deficiency remains unknown. We describe three sisters affected with severe isolated growth hormone deficiency and pituitary hypoplasia caused by biallelic mutations in the *RNPC3* gene, which codes for a minor spliceosome protein required for U11/U12 small nuclear ribonucleoprotein (snRNP) formation and splicing of U12-type introns. We found anomalies in U11/U12 di-snRNP formation and in splicing of multiple U12-type introns in patient cells. Defective transcripts include preprohormone convertases *SPCS2* and *SPCS3* and actin-related *ARPC5L* genes, which are candidates for the somatotroph-restricted dysfunction. The reported novel mechanism for familial growth hormone deficiency demonstrates that general mRNA processing defects of the minor spliceosome can lead to very narrow tissue-specific consequences.

**Subject Categories** Genetics, Gene Therapy ' Genetic Disease; Metabolism

## Introduction

Familial dwarfism with isolated growth hormone (GH) deficiency (IGHD) can be caused by mutations in *GH1* and other genes involved in GH regulation and pituitary development (Pfäffle *et al*, 2011). However, the molecular basis of a significant proportion of cases remains unknown.

Removal by splicing of introns from the primary transcripts of most human genes is an essential step in gene expression. Splicing is performed by spliceosomes, ribonucleoprotein complexes containing numerous proteins and five small nuclear RNAs (snRNAs). Two types of spliceosomes excise distinct classes of introns. The major U2-dependent spliceosome processes most introns, while a small subset (<0.5%) present in approximately 3% of human genes is spliced by the minor U12-dependent spliceosome (Turunen *et al*, 2013). U12-type introns coexist with U2-type introns in the same genes, but are normally spliced at a slower rate (Patel *et al*, 2002). Mutations in the U4atac snRNA component of the minor spliceosome were recently shown to cause a lethal malformation syndrome called microcephalic osteodysplastic primordial dwarfism type 1 (MOPD1) or Taybi-Linder syndrome (TALS) (Edery *et al*, 2011; He *et al*, 2011). Here we report that mutations in a protein component of the minor spliceosome lead to familial IGHD.

## Results

### Case report

**Figure 1:**
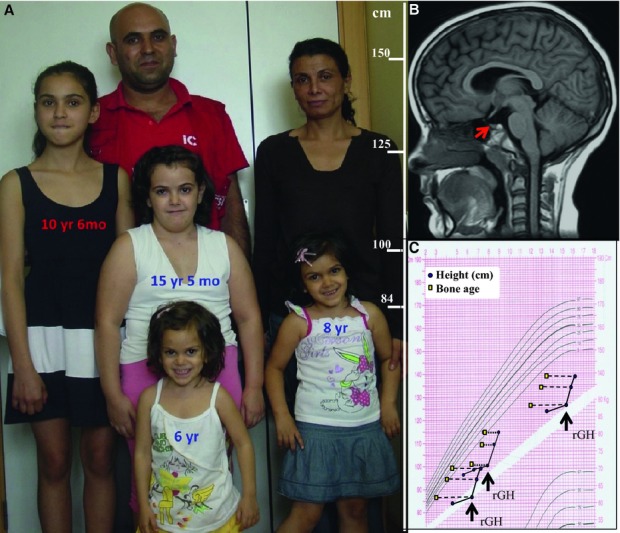
Phenotypic features of probands.
Photograph of family members showing the marked short stature of the three affected girls compared to unaffected relatives, along with other typical features of GHD such as frontal bossing and cherubic face. Ages are shown over each girl, with the height scale on the right.Midline sagittal section of the brain MRI of one proband (IGHD-02). A hypoplastic pituitary is evident (red arrow) with no other brain anomaly.Growth chart including the anthropometric data of the three affected siblings, bone age and height showing delayed and slow basal growth and an excellent response to GH replacement therapy (rGH).

Three sisters born with normal length to normal statured and non-consanguineous parents showed severe postnatal proportionate growth retardation (height −5 to −6.6 SDS at diagnosis), typical physical features of GH deficiency including delayed bone maturation without bone dysplasia, mild microcephaly (−1.1 to −3.1 SDS for height) and otherwise normal development (Fig 1A). GH levels after standard stimuli and basal IGF-I and IGFBP-3 levels were almost undetectable. Prolactin levels were in the low-normal range and the remaining pituitary hormone levels were normal. Brain MRI scans showed hypoplasia of the anterior pituitary (Fig 1B). Total ghrelin levels were extremely elevated with normal acylated ghrelin levels. No other biochemical abnormalities were found. A final diagnosis of familial IGHD with associated pituitary hypoplasia was made. The therapeutic response to GH replacement is excellent to date (Fig 1C), with total ghrelin levels decreasing in all patients (27–66% of values at diagnosis; Supplementary Table 1).

### Mutation identification and validation

**Figure 2:**
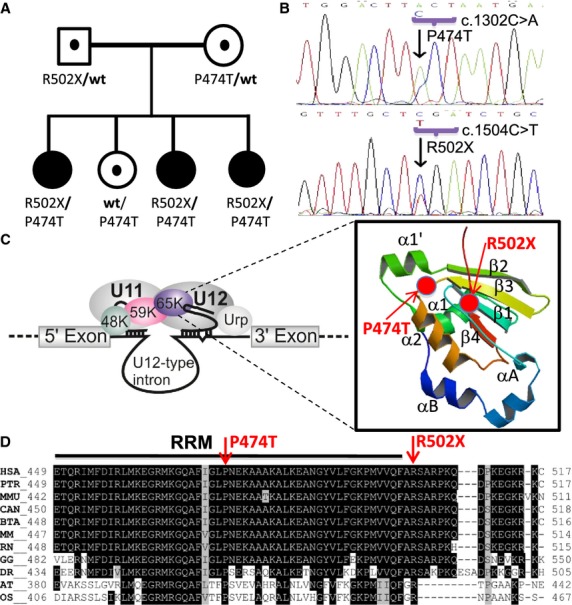
Mutation detection and predicted effects on minor spliceosome.
Family pedigree showing that all three affected girls are compound heterozygotes for the *RNPC3* mutations shown under each symbol while the unaffected parents and sister are heterozygous carriers for one mutation.Sequencing of the RT-PCR product of *RNPC3* from blood RNA of the eldest proband showing the p.P474T (top) and p.R502X mutations in the same amplicon, with relatively decreased expression of the non-sense carrying allele (p.R502X).Schematic representation of the function of the *RNPC3* gene product, U11/U12-65K protein, in RNA splicing. The 65K protein is part of a molecular bridge that links U11 and U12 snRNPs in the intron recognition complex. The mutations may disturb binding of U12 snRNA to 65K protein. Inset indicates the positions of the mutations (red circles) in the 3D structure of the second RRM of the 65K protein determined by X-ray crystallography (Protein Data Bank Identification, PDB-ID: 3EGN).Sequence alignment of the 65K protein second RRM of multiple species using ClustalW algorithm. The mutated residues (red arrows) are highly conserved phylogenetically. (HSA: *Homo sapiens*; PTR: *Pan troglodytes*; MMU: *Macaca mulatta*; CAN: *Canis lupus*; BTA: *Bos taurus*; MM: *Mus musculus*; RN: *Rattus norvegicus*; GG: *Gallus gallus*; DR: *Danio rerio*; AT: *Arabidopsis thaliana*; OS: *Oryza sativa*).

Segregation and mutational analyses discarded involvement of all classical genes of the GH axis (Pfäffle *et al*, 2011). Reverse transcription-polymerase chain reaction (RT-PCR) indicated normal amount and sequence of *GH1* transcripts. Exome analysis of one proband revealed missense (c.1320C>A, p.P474T) and nonsense (c.1504C>T, p.R502X) mutations in the *RNPC3* gene. Sanger sequencing validated that the three affected sisters are compound heterozygous for both mutations. The father is a heterozygous carrier of the p.R502X mutation and the mother and unaffected sister heterozygous carriers of the p.P474T mutation (Fig 2A). *RNPC3* codes for the 65 kDa protein of the minor spliceosome. It binds to the 3′-stem-loop of U12 snRNA and is essential for integrity of U11/U12 di-snRNP that functions in U12-type intron recognition (Benecke *et al*, 2005; Turunen *et al*, 2013). RT-PCR and sequencing of *RNPC3* transcripts from patient cell RNA showed the mutated alleles to be expressed in a 2:1 (maternal:paternal) ratio, likely due to partial nonsense-mediated decay (NMD) of paternal mRNA (Fig 2B). Both mutations are located in the second RNA recognition motif (RRM). The p.P474T mutation alters a highly conserved proline residue located in a turn position between β3-strand and α2-helix (Fig 2C). Such turn positions are typically not replaceable by other amino acids (Betts ' Russell, 2003). In addition to mRNA instability due to NMD, the p.R502X mutation deletes the last 15 C-terminal residues that are highly conserved (Fig 2D). Due to the conservation of the affected residues and their position within or near the C-terminal RRM, both mutations are predicted to have deleterious effects on RNPC3 function, impairing binding to U12 snRNA that could affect U11/12 di snRNP complex stability (Netter *et al*, 2009).

### Minor spliceosome assembly dysfunction in lymphoblastoid cells

**Figure 3:**
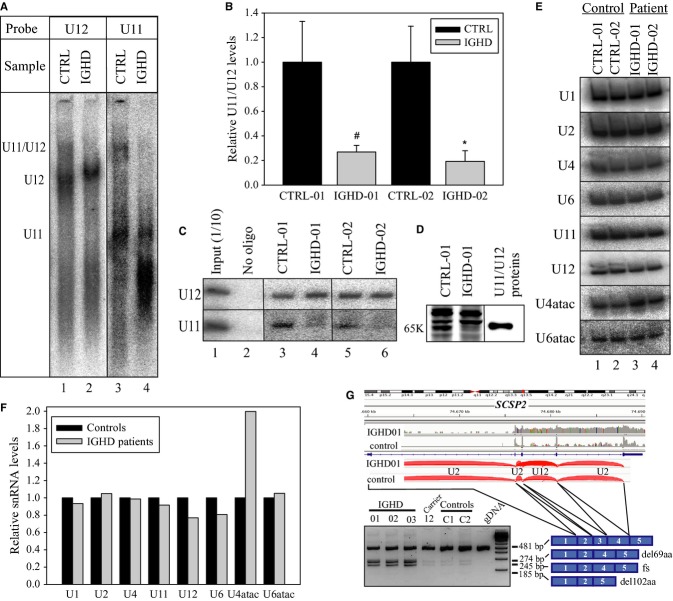
U11/U12 di-snRNP stability, snRNA levels and U12-type splicing efficiency in cells from IGHD patients and controls.
Native gel analysis of U11 and U12 snRNP complexes in nuclear extracts.U12 snRNP pull-down assay measuring the level of U11 snRNP associated with U12 snRNP. Three technical replicates were used for each pulldown. Student's *t* test, **P = 0.02, ***P = 0.01, *n* = 3.Representative gel of data in panel B.Western blot analysis of control and patient nuclear extracts using antibodies targeted to U11/U12-65K protein. Purified U11/U12 di-snRNP was used as control in lane 3.Northern blot analysis of expression levels of spliceosomal snRNAs in lymphoblastoid cell lines from two patients (IGHD-01 and IGHD-02) and two controls (CTRL-01 = LCL422 and CTRL-02 = LCL452). Total RNA was extracted from each cell line and individual snRNAs detected by Northern blotting.Quantification of the snRNAs in D. Data represents mean values of two patient and two control datasets.Transcription profiles of the *SCPS2* gene. RNAseq and RT-PCR obtained concordant results showing relatively poor U12-type splicing in patients with increased intron retention (U12 and flanking U2 introns), along with alternative (aberrant) U2-type splicing (˜30% of transcripts). The alternative transcripts are barely present in controls or heterozygous carriers. The band in genomic DNA lane (gDNA) is derived from a processed pseudogene on chromosome 1. Verification of the appropriate content (real processed mRNA in the cDNA products and processed pseudogene in the genomic amplification) was performed by sequencing. Source data are available for this figure.

Native gel analysis of nuclear extracts revealed nearly complete loss of the functional U11/U12 di-snRNP complex in patient cells (Fig 3A, lanes 3,4) and appearance of a U12 snRNP complex with slightly reduced mobility (Fig 3A, lanes 1,2; Supplementary Fig 11a). Reduced stability of the U11/U12 complex was confirmed in pulldown experiments (Fig 3B,C) and glycerol gradient analyses (Supplementary Fig 11b) using whole cell extracts. Western blot analysis of nuclear extracts showed a significant reduction of 65K protein levels in IGHD patients (Fig 3D).

Analysis of spliceosomal snRNA expression levels in lymphoblastoid cells by Northern blotting revealed that, except for an unexpected twofold increase in U4atac snRNA levels, the snRNA levels of both minor and major snRNPs are largely unaffected (Fig 3E,F). These results indicate that these mutations lead to significant destabilization of the U11/U12 di-snRNP complex in the patient cells.

### Differential splicing defects of minor introns in blood cells

To test the effect of the observed U11/U12 di-snRNP complex destabilization on splicing of U12-type introns, we analyzed RNAseq data in cells from patients and controls. Of the 695 genes listed in the U12DB harboring U12-type introns (Sheth *et al*, 2006; Alioto, 2007), 522 (75%) were detected at sufficient levels to allow comparisons of cases and controls.

We quantified the splicing efficiency and intron retention of U12-type introns with respect to U2-type introns per gene, normalized by gene expression and established a list of 21 genes with significantly decreased U12/U2 ratios in patient cells (Supplementary Table 2). We also observed aberrant processing events including exon skipping and activation of cryptic U2-type splice sites (Fig 3G, Supplementary Figs 1–10).

Splicing efficiency of 15 genes was studied by RT-PCR (Supplementary Table 3). Consistent with RNAseq data, RT-PCR validated the relative increase in abnormal processing of U12 introns in patient cells. The relative levels of unspliced or aberrantly spliced U12-type introns varied from <5 to 30% of the spliced mRNA level.

### Defects in genes related to pituitary-specific expression

A subset of genes with aberrant U12-type intron processing in the IGHD patients encodes proteins with relevant functions in pituitary development, thus becoming candidates for the phenotype. *SPCS2* encodes an inferred protein subunit of the signal peptidase complex implicated in posttranslational processing of preprohormones, including preproghrelin processing to proghrelin (GO: 0005787; Yin *et al*, 2009). Two novel transcripts, representing approximately 30% of *SPCS2* mRNAs in IGHD cases but barely detected in controls, result from partial activation of cryptic U2-type splice sites instead of normal U12-splicing of the third intron (Fig 3G). One transcript encodes a protein with 69 amino acids deleted in the functional domain. The other has a frameshift leading to premature truncation or NMD. Slightly increased intron retention and alternative transcripts were also observed in the *SPCS3* gene that encodes another subunit of the same complex (Supplementary Fig 1).

*ARPC5L* encodes an Arp2/3 complex protein that participates in actin polymerization and is expressed in embryonic anterior pituitary (Ma *et al*, 2009). Defective processing of the U12-type intron leads to activation of nearby cryptic U2-type sites, almost exclusive to IGHD patients, and a transcript encoding a protein with an internal deletion of 27 amino acids (Supplementary Fig 2).

## Discussion

Pituitary development depends on a complex cascade of transcription factors and signaling molecules that dictate organ commitment, cell differentiation and cell proliferation. The most common consequence of an abnormality in these processes is IGHD, as GH-producing cells constitute 30–40% of anterior pituitary cells. GHD has been linked so far to mutations in genes involved in GH synthesis, function and regulation, or in pituitary development (Pfäffle *et al*, 2011).

We identified biallelic mutations in the *RNPC3* gene coding for the minor spliceosome U11/U12-65K protein as a novel mechanism associated with IGHD and pituitary hypoplasia in three cases from a single family. U11/U12-65K protein levels are strictly regulated (Verbeeren *et al*, 2010), as it is an essential component of the intron-recognition complex (U11/U12 di-snRNP) in the ubiquitous minor spliceosome (Benecke *et al*, 2005) involved in splicing of approximately 700 target genes. The U11/U12-65K protein has dual binding activity, interacting directly with U12 snRNA via its C-terminal RRM and with the U11-associated 59K protein via its N-terminus (Will ' Lührmann, 2005; Turunen *et al*, 2013). These interactions are necessary for correct di-snRNP structure formation and intron recognition (Frilander ' Steitz, 1999; Benecke *et al*, 2005). The mutations found here affect the second RRM motif of the 65K protein with consequential defects in U11/U12 di-snRNP assembly and splicing of a subset of U12-type introns, indicating that minor splicesome dysfunction can lead to pituitary hypoplasia and GHD.

These are the first reported patients with GHD due to aberrant mRNA processing, as well as the first pathology described to be associated with a mutation in a protein of the minor spliceosome. It is, however, the second example of a minor spliceosome component associated with human disease. Mutations in the U4atac snRNA component cause the severe form of MOPD1/TALS (Edery *et al*, 2011; He *et al*, 2011). In this disease, usually lethal in early childhood, patients have short bowed long bones, cerebral cortex malformations, dry skin and sparse hair. The phenotype of patients with *RNPC3* mutations is much less severe as growth failure, although severe, was postnatal. Brain size was spared with no malformation except for pituitary hypoplasia, and psychomotor development was normal with no other organ failure. Interestingly, U4atac snRNA levels were elevated in IGHD cells, suggesting a possible compensatory, yet hitherto unknown, mechanism to reduce the effect of the mutations.

Given the ubiquitous function of the minor spliceosome in all cell types, the pathological differences between IGDH and MOPD1/TALS, particularly the tissue-specificity, most likely relate to differences in splicing defect severity. Transcriptome data is unavailable for MOPD1/TALS, but the underlying defect is suggested to be perturbation of multiple transcripts containing U12-type introns that regulate global cell proliferation (Edery *et al*, 2011; He *et al*, 2011). In contrast, the defect in the IGHD patients lead to a relatively low level of aberrant splicing for most genes containing U12-type introns. It is likely that the mild defects in splicing are tolerated in most tissues but not during pituitary development, as somatotrophs are specifically affected. Genes involved in pituitary development that show processing defects in patient cells include the signal peptidases (*SPCS2* and *SPCS3*) and *ARPC5L*.

Similar tissue-specific effects occur in other splicing-related diseases. Spinal muscular atrophy and some forms of retinitis pigmentosa display tissue-specific phenotypes due to mutations affecting both spliceosomes and the processing of numerous transcripts in all cell types (Boulisfane *et al*, 2011; Linder *et al*, 2011). These tissue-restricted effects could result from defective splicing events of a few specific genes or from differential tissue sensitivity to the aberrant processing of numerous transcripts (Cooper *et al*, 2009).

While individuals with complete GHD caused by loss of function *GH1* gene mutations often develop antibodies to exogenous GH (Pérez Jurado *et al*, 1997; Pfäffle *et al*, 2011), patients with *RNPC3* mutations showed excellent response and immune tolerance to GH replacement. The explanation may be that the *GH1* gene is intact, displaying normal expression and processing in blood cells despite the lack of pituitary GH secretion.

In summary, our findings provide novel insights into the molecular basis of a new form of IGHD and demonstrate a crucial role of the minor spliceosome in the processing of genes required for pituitary development and GH synthesis.

## Materials and Methods

### Genetic studies

The study was approved by the Hospital Infantil Universitario Niño Jesús research ethics board and parents provided written informed consent. This study complied with the WMA Declaration of Helsinki and NIH Belmont report.

Deletions at the *GH1* locus were discarded by previously established methods (Pérez Jurado *et al*, 1997). To perform linkage and segregation analysis with known loci involved in GH deficiency, we genotyped two microsatellites per locus (located at <500 Kb) in all six nuclear family members. DNA was amplified using standard conditions and a labeled PCR primer. PCR products were electrophoresed in an ABI PRISM 377 automated sequencer and analyzed with fragment analysis software (Life Technologies). Genotyped loci were: D17S944 and D17S949 (for *GH1*), D20S870 and D20S107 (for *GHRH*), D7S632 and D7S526 (for *GHRHR*), D3S671 and D3S2386 (for *POU1F1*), D5S2008 and D5S2073 (for *PROP1*).

We then used whole-exome sequencing to search for mutations in the DNA sample of proband IGHD1-01 using the Nimblegene 44 Mb capture kit and Illumina HiSeq 2000 sequencing. Sequences were aligned to the NCBI build 37 human genome reference using BWA. Duplicates were removed using Picard. Alignments were recalibrated using GATK. Lane-level indel realignments and base alignment quality adjustments were applied. In addition to the publicly available 1000 genomes (www.1000genomes.org) and the 6503 samples from the exome variant server (gs.washington.edu/evs), we checked exome data from 300 Spanish individuals to confirm the absence of the mutations in all the controls. PCR with exon specific primers and Sanger sequencing of the amplicons were performed for validation. Primer sequences are available upon request.

For functional and expression studies, a lymphoblastoid cell line was generated from the two older affected sisters using standard protocols. Total RNA was isolated from mononuclear blood cells and cell lines using standard methods.

### Analysis of snRNAs and U11/U12 snRNPs

Integrity of U11/U12 di-snRNP complexes was analyzed using native gel analyses (Frilander ' Meng, 2005), glycerol gradient sedimentation of lymphoblastoid cell lysates (Will *et al*, 2004) and via pull-down assays. Nuclear lymphoblastoid cell extracts were prepared as described earlier (Dignam *et al*, 1983). Whole cell extracts from the same cells were prepared by dissolving cell pellets in non-denaturing lysis buffer (20 mM tris-HCl, pH 8; 137 mM NaCl; 10% glycerol; 1% NP40; 2 mM EDTA), followed by sonication on ice and clearing by centrifugation (16 000 *g*, 30 min). The resulting supernatant was loaded on the top of a 10–30% glycerol gradient in buffer (20 mM Hepes, pH 7.9; 100 mM KCl; 1 mM MgCl_2_) and centrifuged (Sorvall TH641, 210 000 *g* (average), 16 h, +4°C) followed by fractionation. Alternatively, the whole cell lysates were incubated with 50 pmol biotinylated 2′-O-methyl RNA oligonucletide complementary to U12 snRNA in 50 mM Hepes-KCl, pH 7.9, 100 mM KCl, 20 mM CRP, 0.5 mM ATP, 3 mM MgCl_2_, followed by pull-down with 25 μl of streptavidin beads. After washing five times with 20 volumes of washing buffer (50 mM Hepes-KCl, pH 7.9, 100 mM KCl, 3 mM MgCl_2_), the RNAs were isolated from pellet fractions. Northern blot analysis was carried out as described earlier (Pessa *et al*, 2006; Verbeeren *et al*, 2010). The data were collected by phosphoimaging (Fuji FLA-5010) and analyzed with AIDA software (Raytest, Germany).

### Semi-quantitative RT-PCR analysis

Splicing efficiency of U12-type introns and alternative processing was tested by RT-PCR analysis of total RNA. Total RNA from mononuclear blood cells was treated with RQ1 RNase free DNase (Promega). First-strand cDNAs were synthesized from 2 μg of total RNA using random hexamers and SuperScript II RT (BioRad). One-tenth of the reaction was used in PCR [EcoTaq polymerase (Ecogen); 1x Taq buffer containing 20 pmol of primers, 200 μm dNTP, 1.5 mm MgCl_2_; 30 cycles]. We used intron-specific and exon-spanning primers to quantify pre-mRNAs/mRNAs in which the intron under investigation was either retained or spliced, respectively. Flanking U2-type introns were included in each gene as an internal reference. Primer sequences and PCR regimes and regions are shown in Supplementary Table 3. The PCR products were separated on 1.5–3.5% agarose gels containing ethidium bromide and visualized under UV light. The gel images were digitally captured and analyzed using BioRad software.

### Transcriptome analyses

RNAseq was obtained from total RNA from mononuclear blood cells of two patients (IGHD-01 and 02) and four control individuals of normal stature and with no endocrine anomalies, by using Illumina HiSeq 2000 sequencing generating paired reads 100 bp in length. Sequences were aligned to the NCBI build 37 human genome reference using Bowtie 2 (http://bowtie-bio.sourceforge.net/bowtie2/index.shtml). TopHat 2.0.4 (http://tophat.cbcb.umd.edu) was used to map the inter-exon splice junctions. Cufflinks (http://cufflinks.cbcb.umd.edu) was used to assemble the aligned RNAseq reads into transcripts and estimate their abundance by quantifying the Fragments Per Kilobase of transcript per Million fragments mapped (FPKM value).

On average, 41.8 million mapped reads were obtained in cases and 27.8 million reads in control samples. The integrative genome viewer (IGV) application was used to visualize the data mapped by Bowtie 2 and TopHat. Relative splicing efficiency of the U12-type introns of the human genome was then quantified in cases and controls (881 introns in 695 genes annotated in the U12 database, U12DB, http://genome.crg.es/datasets/u12) (Alioto, 2007). We quantified the split reads across exon junctions processed by U12 splicing, normalized with respect to the split reads across exon junctions processed by two U2 splicing on the same genes and with respect to the FPKMs per gene. Average U12/U2 and U12/FPKM ratios per gene were established in each patient and control sample. Intron retention was also quantified by examining the depth coverage of U12 intronic reads normalized with respect to the depth coverage of two U2 intronic positions of the same gene as above and with respect to the FPKMs per gene. Genes with high variability in controls were discarded. By comparing cases and controls, a final rank of genes showing significantly different values (above 2 standard deviations) in cases in at least two of the previous analyses was established.

**The paper explained**

**Problem**

Proportionate short stature due to growth hormone (GH) deficiency is well identified both clinically and auxologically. Although a number of patients exhibit abnormalities in the gene coding for GH itself (*GH1*) or for the GHRH receptor (*GHRHR*) resulting in isolated GH deficiency, or in genes involved in pituitary development, such as *POUF1*, *PROP1*, *LHX3* and *LHX4*, resulting in combined pituitary hormone deficiencies, the genetic etiology has not been described in a number of patients. We report here a family with four daughters, three of them affected with severe short stature due to isolated GH deficiency and with pituitary hypoplasia. No abnormality was found in any of the genes known to be involved in either isolated GH deficiency or combined pituitary hormone deficiencies.

**Results**

Compound heterozygous mutations in the gene *RNPC3* involved in the formation of the minor spliceosome were found in all three affected girls. This resulted in anomalies in minor spliceosome formation and incorrect and/or incomplete splicing of a variety of mRNAs in patient cells, including some genes coding for proteins involved in the development of the anterior pituitary.

**Impact**

This is the first time that mutation of a protein of the minor spliceosome has been implicated in patients with isolated GH deficiency. This finding could explain some of the cases of severe GH deficiency that have yet to receive a molecular diagnosis. Our results also indicate that variations in specific protein components of the spliceosomes could underlie other pathological entities.
