# Supplementary material for: Defective minor spliceosome mRNA processing results in isolated familial growth hormone deficiency
Source: EMBO Mol Med. 2014 Jan 30;6(3):299–306. doi: 10.1002/emmm.201303573 (PMC3958305; doi:10.1002/emmm.201303573)
Supplement: Supplementary file 1 [file emmm0006-0299-sd1.pdf]

Defective minor spliceosome mRNA processing results in isolated familial growth hormone deficiency

Jesús Argente, Raquel Flores, Armand Gutiérrez-Arumí, Bhupendra Verma, Gabriel A. Martos-Moreno, Ivon Cuscó, Ali Oghabian, Julie A. Chowen, Mikko J. Frilander, Luis A. Pérez-Jurado

Corresponding authors:

Jesús Argente, Hosptial Infantil Universitario Niño Jesús, Universidad Autonoma de Madrid
Luis A. Pérez-Jurado, Genetics Unit, Universitat Pompeu Fabra
Mikko J. Frilander, Institute of Biotechnology, University of Helsinki

Review timeline:

Submission date:	14 October 2013
Editorial Decision:	07 August 2013
Additional Author Correspondence :	09 August 2013
Additional Editorial Correspondence:	09 August 2013
Resubmission:	14 October 2013
Editorial Decision:	19 November 2013
Revision received:	27 November 2013
Accepted:	02 December 2013

Transaction Report:

(Note: With the exception of the correction of typographical or spelling errors that could be a source of ambiguity, letters and reports are not edited. The original formatting of letters and referee reports may not be reflected in this compilation.)

Editor: Roberto Buccione

1st Editorial Decision 07 August 2013

Thank you for the submission of your manuscript to EMBO Molecular Medicine. We have now heard back from the three Reviewers whom we asked to evaluate your manuscript.

As you will see, all three Reviewers independently point to similar issues that altogether preclude publication of the manuscript in EMBO Molecular Medicine. I will not discuss the evaluations in detail, as they are clear. The main point is that the Reviewers appear to agree that the main conclusion (and interpretation) is not supported by the data presented.

I am sure you will understand that all considered, the Reviewers' concerns are too fundamental and thus leave us no choice but to return the manuscript to you. In our assessment it is not realistic to expect to be able to address these issues experimentally and to the satisfaction of the Reviewers in a reasonable time frame.

I am sorry to disappoint you on this occasion. I hope, however, that the Reviewers' comments will be helpful for your continued work in this area.

***** Reviewer's comments *****

Referee #1 (Remarks):

In this manuscript the authors describe a mutation in a subunit of minor spliceosome in a family with isolated growth hormone deficiency. The authors report that there are anomalies in U11/U12 di-snRNP formation and splicing of multiple U12-type introns.

Major comments

The functional defects reported by the authors are not convincing. The supposed minor spliceosome assembly dysfunction, based upon the experiments in Fig 3., are not obvious by inspection of the data and, in any case, are very subtle.

In the splicing experiments shown in the supplementary figures, in some cases new products are observed in the affected patient samples but it appears that correctly spliced product is present at normal (or near normal) levels. No experiments are performed to examine protein levels. For these reasons, it is not clear that aberrant splicing is the cause of the disease. Moreover, it is not clear from the presented data which mRNA/protein would be responsible for the disease.

Referee #2 (Remarks):

This study reports mutations in a protein subunit of the U11/U12 minor snRNP in three sisters with a form of isolated familial growth hormone deficiency. The patients are compound heterozygotes for nonsense and missense mutations in the RNPC3 gene. The work is of interest because it is the first example of a disease-associated mutation affecting a protein subunit of the minor spliceosome. (Mutation of the U4atac snRNA was previously reported, and has a different and more severe phenotype). There are only ~700 minor introns in the human genome, and it is of interest that the phenotype is relatively restricted. The authors identify multiple splicing defects in patient blood cells by RNA-seq and RT-PCR, including in genes involved in pituitary gland development, potentially accounting for the disease phenotype.

The study is relatively straightforward, but there is room for improvement, as indicated below.

1. Introduction - "mutually exclusive classes of introns" may be confusing to some readers not well versed in splicing mechanisms. Both types of introns usually coexist in certain genes, as mentioned later. I suggest "distinct classes of introns".

2. The data in Figure 3A,B are only suggestive, as the snRNP sedimentation differences between patients and controls appear small, and the figure only shows one representative gel and the average of two patients versus two controls. It's difficult to see what the variability in the measurements is, unless more replicates, standard deviations, and statistical significance are shown.

3. Figure 3C: In this case there are error bars, but I could not find information regarding the number of replicates or whether the difference is statistically significant.

4. Figure 3D,E: Once again, it would be highly desirable to increase the number of replicates to at least n=3 and to provide standard deviations and statistical significance. The reduction in U11 and U12 looks minimal, and may or may not exceed normal variation. The increase in U4atac appears more striking, although it is not known whether and how it arises from the RNPC3 defect, as this protein is not associated with U4atac snRNA.

5. It would be of interest to know whether there is a reduction in U11/U12 snRNA and snRNP levels, or an increase in U4atac snRNA, in cells from the unaffected heterozygote parents or sister, if these are available.

6. If space permits, one of the RT-PCR validation results, e.g., Fig S1, could be shown as a main figure.

7. Again, if space permits, it would seem worthwhile to mention that the RNPC3 gene itself has a U12-dependent intron, although it did not show abnormal splicing in patient blood cells (from Table S3).

Referee #3 (Remarks):

Argente et al. identify a family of with four children, three of which are deficient in growth hormone and show significant post-natal growth retardation. All affected children have biallelic mutations in the RNPC3 gene which encodes a protein specific for the minor spliceosome. The authors investigate the hypothesis that the U12-type spliceosomal splicing pathway is affected in cells from these patients using gradient separation of snRNP complexes and RNA-Seq analysis of splicing efficiency and patterns. They identify a number of genes with affected U12-type introns, three of which have possible connections to the hypopituitary phenotype.

In general, this is an interesting description of a new potential phenotype caused by disruption of core splicing factors. However, a few concerns are raised below where the depth of analysis could be improved with a few more experiments.

The major conclusion of the paper is somewhat undermined by the rather small effects on U11/U12 di-snRNP levels and snRNA levels. No direct measurements of RNPC3 protein levels or mRNA levels are presented. Commercial antibodies appear to be available. This protein is known to be regulated through feedback mechanisms which might be expected to provide compensation. The family data suggest that neither allele alone is sufficient to cause disease.

It would strengthen the proposed link between the RNPC3 mutations and the RNA-Seq data showing reduced or aberrant U12-type intron splicing if the authors expressed wild type RNPC3 protein in their mutant cells and showed restoration of wild type splicing by RT-PCR. Alternatively or additionally, an RNAi knockdown of wild type RNPC3 should be done to show that the splicing defects can be recapitulated.

Minor point:

The last sentence of the abstract suggests a novel finding that "general gene splicing defects can lead to narrow tissue-specific defects." This is clearly not a novel result since it also applies to adRP and SMA as two examples. The sentence should be reworded.

Additional Author Correspondence 09 August 2013

Thank you for your consideration of our manuscript EMM201303284. We understand that the manuscript is rejected in its current form. We think that the comments by the referees are constructive and believe that many of their concerns can be adequately address in a reasonable period of time. Indeed, some of the additional data requested has already been generated.
Hence, I would like to ask whether you would be willing to reconsider this manuscript if we were to resubmit it, taking into consideration the concerns of the referees.

Thank you for your time and I am looking forward to hearing from you soon.

Additional Editorial Correspondence 09 August 2013

Thank you for your letter.

Considering the potential interest of your findings, we would have no objection to consider a new

manuscript on the same topic if at some time in the near future you have obtained data that would considerably strengthen the message of the study and address the Reviewers' concerns.

Please consider, however, that if you were to send a new manuscript this would be treated as a new submission rather than a revision and would be reviewed anew.

If you should decide to do so, please mention this in your cover letter and provide a detailed point-by-point rebuttal of the Reviewers' evaluations on your original version.

Resubmission 14 October 2013

RESPONSE TO REVIEWERS COMMENTS

First of all, we wish to thank all three reviewers for their encouraging and critical comments that led us to improve our methodology and significantly improve this manuscript.

Referee #1 (Remarks):

In this manuscript the authors describe a mutation in a subunit of minor spliceosome in a family with isolated growth hormone deficiency. The authors report that there are anomalies in U11/U12 di-snRNP formation and splicing of multiple U12-type introns.

Major comments

Q: The functional defects reported by the authors are not convincing. The supposed minor spliceosome assembly dysfunction, based upon the experiments in Fig 3., are not obvious by inspection of the data and, in any case, are very subtle.

R: We fully agree with the reviewer that the observed biochemical differences are relatively mild and perhaps hard to appreciate using the methods used in the study. In fact, we believe that these mild biochemical defects are real and somehow explain why the phenotype in these patients is not severe, with the secondary splicing defects only having consequences in a single tissue. However, to address the concerns of this referee we have now carried out additional analyses to strengthen the biochemical part:

In effect, we have now analyzed the U11/U12 di-snRNP integrity on nuclear extract preparations using native gel analysis. This system provides a better resolution of the individual snRNP complexes than the glycerol gradient analysis, where the different complexes migrate very close to each other and are partially overlapping. The native gel analysis now provides a striking contrast between patient (IGHD1) and control cells with virtually no U11/U12 di-snRNPs detected in the IGHD1 cells. This is in particularly evident with plots probed for U11 snRNA. The results are also very consistent with our gradient analysis, which also showed that a minor fraction of U11 was in the U11/U12 complex even in control cells.

This result led us to re-examine the results of the pull-down analysis as well, since U11 snRNP levels in the control cells were higher than those observed in gradient analyses and native gels. We found that the bead washing steps in the original analyses were insufficient, resulting in increased levels of U11 snRNA both in control and patient cells after the pulldown, which was partially masking the level of the defect. Using the extended washing conditions (with increased washing buffer volume only and no other modifications) and analyzing the two patient cells separately we now find a significant defect between the control and patient cells. The magnitude of the defect is now consistent between the three different analyses, i.e. our original glycerol gradient, the native gel and the pull-down analyses. The native gel also provides an explanation of the apparently small effect of the IGHD1 mutation in U12 sRNP gradient sedimentation (U12 mono-snRNP vs U11/U12 di-snRNP) in patient cells: the resulting mono-U12 snRNP has an intermediate mobility that most likely migrates very close to the U11/U12 di-snRNP peak and thus prevents accurate quantification of the glycerol gradient fractions.

As a result of the new data, we have now reorganized Figure 3 so that only native gel and pulldown analyses are shown. The gradient data are moved to the supplementary section and additional native gel data, a heparin titration analysis that further supports the native gel analysis, are included in the supplementary section as well. Finally, a panel of splicing analysis requested by referee 3 is added to Figure 3.

Q: In the splicing experiments shown in the supplementary figures, in some cases new products are observed in the affected patient samples but it appears that correctly spliced product is present at normal (or near normal) levels. No experiments are performed to examine protein levels. For these reasons, it is not clear that aberrant splicing is the cause of the disease. Moreover, it is not clear from the presented data which mRNA/protein would be responsible for the disease.

R: The reviewer is correct that, in addition to increased amount of aberrantly spliced products, the normal mRNA products are always observed in the patients' samples for all the U12-processed genes tested. Similar cases in which only very minor (quantitative) defects were observed for U12-type intron splicing, but which nevertheless led to the death of organisms/children have been observed earlier by us (Pessa, et al PLoS ONE 5, e13215) and others (Edery, et al. Science 332:240-243; He, et al. Science 332:238-240). In this case the disease pathology is very mild and therefore, mild splicing effects are expected. If stronger defects were observed, then the question would be why the observed defect is not similar to the earlier characterized MOPD1/TALS disorder with severe malformations and death at the age of 3. We now stress this issue in the revised manuscript.

Related to the question of which of the genes are responsible for the disease: We provide several tentative possibilities that we uncovered by the analysis of mRNA patterns in blood lymphocytes. One should, however, bear in mind that lymphocytes are not the affected tissue (which we cannot sample for practical and ethical reasons) and therefore firm causal relationships are difficult to attain with this analysis. Other than the different hormones and growth factors that we analyzed in circulating plasma of the probands by RIA (GH, TSH, IGF1, ghrelin,...), there are no candidate genes suitable for protein studies in the available tissue.

Referee #2 (Remarks):

This study reports mutations in a protein subunit of the U11/U12 minor snRNP in three sisters with a form of isolated familial growth hormone deficiency. The patients are compound heterozygotes for nonsense and missense mutations in the RNPC3 gene. The work is of interest because it is the first example of a disease-associated mutation affecting a protein subunit of the minor spliceosome. (Mutation of the U4atac snRNA was previously reported, and has a different and more severe phenotype). There are only ~700 minor introns in the human genome, and it is of interest that the phenotype is relatively restricted. The authors identify multiple splicing defects in patient blood cells by RNA-seq and RT-PCR, including in genes involved in pituitary gland development, potentially accounting for the disease phenotype.

The study is relatively straightforward, but there is room for improvement, as indicated below.

1. Introduction - "mutually exclusive classes of introns" may be confusing to some readers not well versed in splicing mechanisms. Both types of introns usually coexist in certain genes, as mentioned later. I suggest "distinct classes of introns".

R: We agree with the reviewer and have changed the wording.

2. The data in Figure 3A,B are only suggestive, as the snRNP sedimentation differences between patients and controls appear small, and the figure only shows one representative gel and the average of two patients versus two controls. It's difficult to see what the variability in the measurements is, unless more replicates, standard deviations, and statistical significance are shown.

R: The sedimentation differences are indeed very subtle and partially overlapping between the 18S U11/U12 di-snRNP and 15S U12 mono-snRNP. For gradient analysis we were not able to increase the number of biological replicates as we only had cell lines from two patients available. To clarify this issue we decided to use another method, a native gel analysis, to clarify the status of the

U11/U12 di-snRNP integrity. This method uses nuclear extracts and has a better resolution in separating the individual snRNP complexes.

As described in our response to referee 1 above, the new analysis now provides a significant difference between the control and patient cells, with virtually no U11/U12 complex present in the patient cells (particularly evident in figure 3, the panel probed for U11 snRNA (lanes 3 and 4) and in supplementary figure S11). Also, as described above, with U12 snRNA probing we noticed an aberrant complex with an intermediate mobility between U12-mono snRNP and U11/U12 di-snRNP. This complex also explains the relatively poor resolution of the glycerol gradient analysis earlier. Therefore, given the poor separation of the different gradient fractions, we opted rather to show the native gel analysis and new pulldown data in the main figure.

Q: Figure 3C: In this case there are error bars, but I could not find information regarding the number of replicates or whether the difference is statistically significant.

R: As described above in our response to referee 1, the pulldown experiment now uses extended washing conditions and provides similar results as in the native gel analyses. The technical replicates were separate cell culture batches (n=3) and they show a statistically significant difference between control and patient cells. We have also now included a sample gel of the pulldown analyses to accompany the analysis.

Q. Figure 3D,E: Once again, it would be highly desirable to increase the number of replicates to at least n=3 and to provide standard deviations and statistical significance. The reduction in U11 and U12 looks minimal, and may or may not exceed normal variation. The increase in U4atac appears more striking, although it is not known whether and how it arises from the RNPC3 defect, as this protein is not associated with U4atac snRNA.

R: In this case it was not possible to increase the number of biological replicates as we have only two control and two patient cell lines available. Also, the purpose of this figure was to show that there is virtually no change in snRNA levels except for U4atac. We have now changed the wording in the text to emphasize the similarity rather the differences between the cell lines. Also, given that no experimental treatments are done to the cells, it did not seem relevant to carry out technical replication of the RNA isolations from the same cells.

5. It would be of interest to know whether there is a reduction in U11/U12 snRNA and snRNP levels, or an increase in U4atac snRNA, in cells from the unaffected heterozygote parents or sister, if these are available.

R: This is a good suggestion, but unfortunately we do not have such cell lines available. However, given the minor reduction in U11/U12 snRNP and increase in U4atac snRNA observed in the compound heterozygous individuals and the lack of relevant splicing anomalies detected in blood RNA of heterozygous parents as compared to controls (supplementary figures), it is unlikely that these studies would lead to significant changes.

6. If space permits, one of the RT-PCR validation results, e.g., Fig S1, could be shown as a main figure.

R: We now show one of the supplementary figures as a panel in Figure 3.

7. Again, if space permits, it would seem worthwhile to mention that the RNPC3 gene itself has a U12-dependent intron, although it did not show abnormal splicing in patient blood cells (from Table S3).

R: We agree with the referee that it would be very interesting to discuss this topic. Unfortunately, the limited space did not allow this. However, further information regarding RNPC3 is in Supplementary figure 9.

Referee #3 (Remarks):

The major conclusion of the paper is somewhat undermined by the rather small effects on U11/U12 di-snRNP levels and snRNA levels. No direct measurements of RNPC3 protein levels or mRNA levels are presented. Commercial antibodies appear to be available. This protein is known to be regulated through feedback mechanisms which might be expected to provide compensation. The family data suggest that neither allele alone is sufficient to cause disease.

R: We did measure RNPC3 mRNA levels in patients' samples as part of the global RNAseq data, as well as by direct assays by RTPCR, which included the comparative analysis of allelic expression shown in figure 2. Western blot analysis of protein levels is an excellent suggestion. Unfortunately, all the commercial antibodies that we have tested are of poor quality and do not provide specific results in WB analysis using whole cell extract. Here we have used a previously published antibody from the Lührman lab, which works with purified U11/U12 complexes, but partially also with nuclear extracts, with an acceptable level of non-specific bands. This data is now presented as Figure 3d and suggests that there is a significant reduction of U11/U12 protein in the nuclear extract preparation.

It would strengthen the proposed link between the RNPC3 mutations and the RNA-Seq data showing reduced or aberrant U12-type intron splicing if the authors expressed wild type RNPC3 protein in their mutant cells and showed restoration of wild type splicing by RT-PCR. Alternatively or additionally, an RNAi knockdown of wild type RNPC3 should be done to show that the splicing defects can be recapitulated.

R: We agree that the proposed rescue experiment would be an excellent experiment and we have given such experiments a serious attempt already prior to the submission of our manuscript. However, all of our attempts to carry out the rescue experiment failed due to very low transfection efficiencies with the patient and control cells. The proposed RNAi experiments have multiple issues, both conceptual and technical. First, at the conceptual level they would only establish whether RNPC3 is needed for the splicing of minor introns, but would fail to provide any further support for the mutation phenotype. At the technical level, even if it would be possible to transfect the cells with siRNA oligos or plasmids (see above), it has turned out to be impossible to knock down the RNPC3 transcripts in any cell line (Benecke, H., Lührmann, R., and Will, C.L. (2005). EMBO J 24, 3057-3069; our unpublished data).

Minor point:

Q: The last sentence of the abstract suggests a novel finding that "general gene splicing defects can lead to narrow tissue-specific defects." This is clearly not a novel result since it also applies to adRP and SMA as two examples. The sentence should be reworded.

R: We have now reworded the last sentence as suggested.

2nd Editorial Decision 19 November 2013

Thank you for the submission of your revised manuscript to EMBO Molecular Medicine. We have now received the enclosed reports from the referees that were asked to re-assess it. As you will see the reviewers are now globally supportive and I am pleased to inform you that we will be able to accept your manuscript pending the following final minor amendments and provision of information:

1) Reviewer 2 notes an issue related to the data in Fig. 3G. Please provide an explanation and produce corrections were necessary.

2) Reviewer 3 notes an error in the legend to Fig. 3B concerning expression of a P value. In this regard, please allow me to remind you that as per our Author Guidelines, the description of all reported data that includes statistical testing must state the name of the statistical test used to generate error bars and P values, the number (n) of independent experiments underlying each data point (not replicate measures of one sample), and the actual P value for each test (not merely 'significant' or 'P < 0.05').

3) We would need a short list (up to 5 maximum) of bullet points that summarize the key NEW findings. The bullet points should be designed to be complementary to the abstract and will be used online in our new platform (coming January 2014).

4) Finally, we are now encouraging the publication of source data, particularly for electrophoretic gels and blots, with the aim of making primary data more accessible and transparent to the reader. Would you be willing to provide a PDF file per figure that contains the original, uncropped and unprocessed scans of all or at least the key gels used in the figure? The PDF files should be labeled with the appropriate figure/panel number, and should have molecular weight markers; further annotation may be useful but is not essential. The PDF files will be published online with the article as supplementary "Source Data" files. If you have any questions regarding this please contact me.

Please submit your revised manuscript within two weeks. I look forward to seeing a revised form of your manuscript as soon as possible.

***** Reviewer's comments *****

Referee #1 (Remarks):

The authors have made a conscientious attempt to respond to the comments of the three reviewers. Although I consider the demonstration and characterization of the splicing defect to be only marginally convincing, based upon all of the presented results, and understanding the limitations in obtaining patient samples, I would support publication.

Referee #2 (Remarks):

The authors have responded to all previous concerns and supplied new data that more clearly demonstrates the defects they see. While the effects are rather small in many cases, this is probably a consequence of the incomplete nature of the RNPC3 mutations. They are also to demonstrate significant changes in snRNP complexes and splicing alterations in a subset of U12-type intron containing genes.

Minor point: In Figure 3G, the gel has a lane next to the DNA marker that appears to be labeled "gDNA" and has an arrow below it. This is not described in the text or legend. If gDNA means genomic DNA, how does this generate a spliced product band?

Referee #3 (Remarks):

The authors provided a thoughtful rebuttal, and made some changes that improved the manuscript. In some cases, they were not able to provide additional data, due to limited availability of patient cell lines or various technical difficulties.

The main improvement, besides clarifications in the text, is an analysis of the U11/U12 di-snRNP defect using native gels in nuclear extract, versus the previous sedimentation analysis. Also, more stringent washing in the snRNP pulldown makes the defect more readily apparent. In the new Fig. 3B, the statistical analysis is based on technical replicates, i.e., same patient cell lines but grown in different batches. This is not ideal, but I think it is acceptable. Note that the figure legend should read $P<0.01$ instead of $P>0.01$.

In Fig 3F, the authors argued against providing technical replicates, because the cells were not experimentally treated. Since they now emphasize that there appears to be no significant change in U11/U12 levels between the two patient and two control lines, this is probably acceptable. The apparent increase in U4atac, which was unexpected, is what catches the eye in this figure, but it is

hard to conclude much from n=2. However, U4atac is not the focus of the paper.

I would endorse publication, because it is an interesting and novel finding, implicating the minor spliceosome pathway in a genetic disease. On the negative side, it is still the case that the effects are rather subtle, but this is a feature of the disease and the fact that the analysis is necessarily limited to samples from just two patients.

1st Revision - authors' response 27 November 2013

Referee #1 (Remarks):

The authors have made a conscientious attempt to respond to the comments of the three reviewers. Although I consider the demonstration and characterization of the splicing defect to be only marginally convincing, based upon all of the presented results, and understanding the limitations in obtaining patient samples, I would support publication.

Response to Referee #1:

Thank you for your support of this manuscript.

Referee #2 (Remarks):

The authors have responded to all previous concerns and supplied new data that more clearly demonstrates the defects they see. While the effects are rather small in many cases, this is probably a consequence of the incomplete nature of the RNPC3 mutations. They are also to demonstrate significant changes in snRNP complexes and splicing alterations in a subset of U12-type intron containing genes.

Minor point: In Figure 3G, the gel has a lane next to the DNA marker that appears to be labeled “gDNA” and has an arrow below it. This is not described in the text or legend. If gDNA means genomic DNA, how does this generate a spliced product band?

Response to Referee #2:

1. *We agree that many of the effects are rather small, and as described in the text, this is most likely the reason why the consequence is the relatively mild and tissue-restricted phenotype observed.*
2. *We thank the reviewer for noticing this missing information. It was better explained in the previous version where figure 3G was supplementary figure 1 and we had more room for explanation. The amplification from gDNA results from a processed pseudogene, although we demonstrated by gel isolation and sequencing that there was no DNA contamination in the amplicons from cDNA. We have added a sentence in the figure legend and a more detailed explanation in the source data provided as supplementary data.*

Sentence in the figure legend:

3G) Transcription profiles of the SCPS2 gene. RNAseq and RT-PCR obtained concordant results showing relatively poor U12-type splicing in patients with increased intron retention (U12 and flanking U2 introns), along with alternative (aberrant) U2-type splicing (~30% of transcripts). The alternative transcripts are barely present in controls or heterozygous carriers. The arrow shows the amplification of a single band from genomic DNA derived from a processed pseudogene on chromosome 1. Verification of the appropriate content (real processed mRNA in the cDNAproducts and processed pseudogene in the genomic amplification) was verified by sequencing.

Referee #3 (Remarks):

The authors provided a thoughtful rebuttal, and made some changes that improved the manuscript. In some cases, they were not able to provide additional data, due to limited availability of patient cell lines or various technical difficulties.

The main improvement, besides clarifications in the text, is an analysis of the U11/U12 di-snRNP defect using native gels in nuclear extract, versus the previous sedimentation analysis. Also, more stringent washing in the snRNPpulldown makes the defect more readily apparent. In the new Fig. 3B, the statistical analysis is based on technical replicates, i.e., same patient cell lines but grown in different batches. This is not ideal, but I think it is acceptable. Note that the figure legend should read $P<0.01$ instead of $P>0.01$.

In Fig 3F, the authors argued against providing technical replicates, because the cells were not experimentally treated. Since they now emphasize that there appears to be no significant change in U11/U12 levels between the two patient and two control lines, this is probably acceptable. The apparent increase in U4atac, which was unexpected, is what catches the eye in this figure, but it is hard to conclude much from n=2. However, U4atac is not the focus of the paper.

I would endorse publication, because it is an interesting and novel finding, implicating the minor spliceosome pathway in a genetic disease. On the negative side, it is still the case that the effects are rather subtle, but this is a feature of the disease and the fact that the analysis is necessarily limited to samples from just two patients.

Response to Referee #3:

Thank you for your comments.

We have changed the P value in the figure legend. Thank you for pointing this out.

© EMBO
