# Supplementary figures and images for: Defective minor spliceosome mRNA processing results in isolated familial growth hormone deficiency
Source: EMBO Mol Med. 2014 Jan 30;6(3):299–306. doi: 10.1002/emmm.201303573 (PMC3958305; doi:10.1002/emmm.201303573)

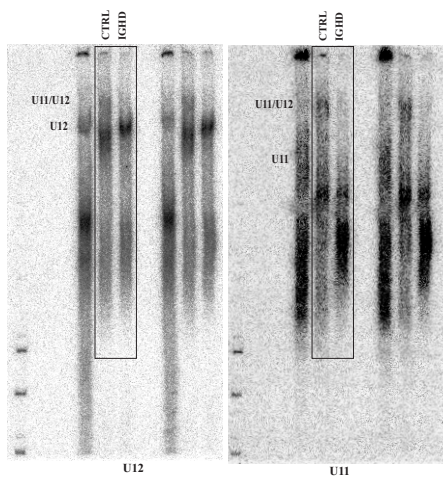

Figure 3A

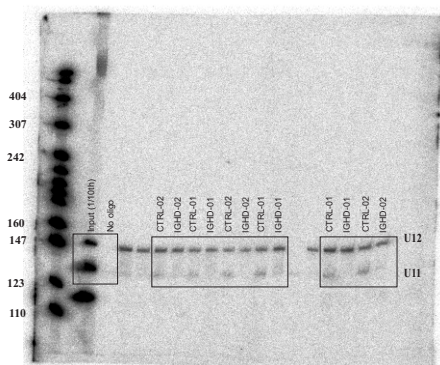

Figure 3C

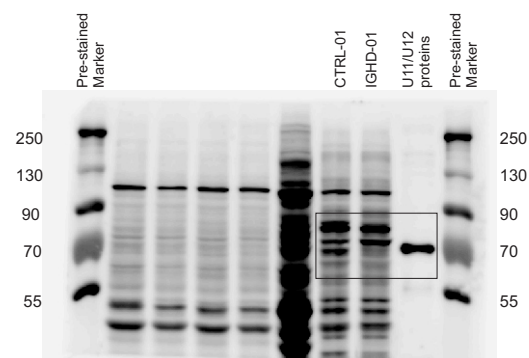

Figure 3D

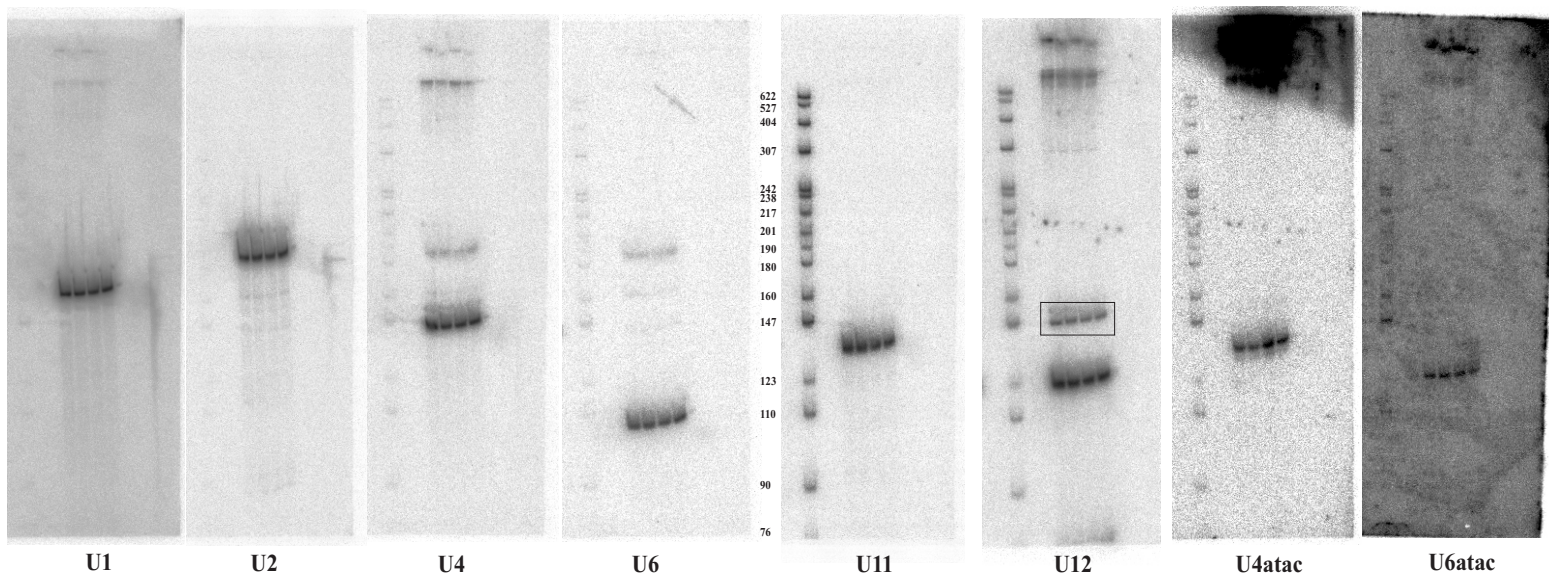

Figure 3E

Supplement: Supplementary file 2 [file emmm0006-0299-sd2.pdf]
