# Supplementary material for: Defective minor spliceosome mRNA processing results in isolated familial growth hormone deficiency
Source: EMBO Mol Med. 2014 Jan 30;6(3):299–306. doi: 10.1002/emmm.201303573 (PMC3958305; doi:10.1002/emmm.201303573)
Supplement: Supplementary file 3 [file emmm0006-0299-sd3.pdf]

Whole image from figure 3g.

Top: Visualization of RNAseq data patterns of whole blood RNA from IGHD patients and controls at the *SPCS2* gene. The upper panel shows the average number of reads (depth coverage) of one patient (IGHD-01) and one control with respect to the genomic structure of the gene (exons represented by blue squares). The lower panel shows the split-read patterns of two patiens (IGHD 01 & 02) along with four controls

Bottom: Expression analysis of the *SPCS2* gene by RT-PCR from whole blood RNA (7 samples). Intact 3% agarose MS-8 gel stained with ethidium bromide: negative image. The predicted transcripts are represented on the right.

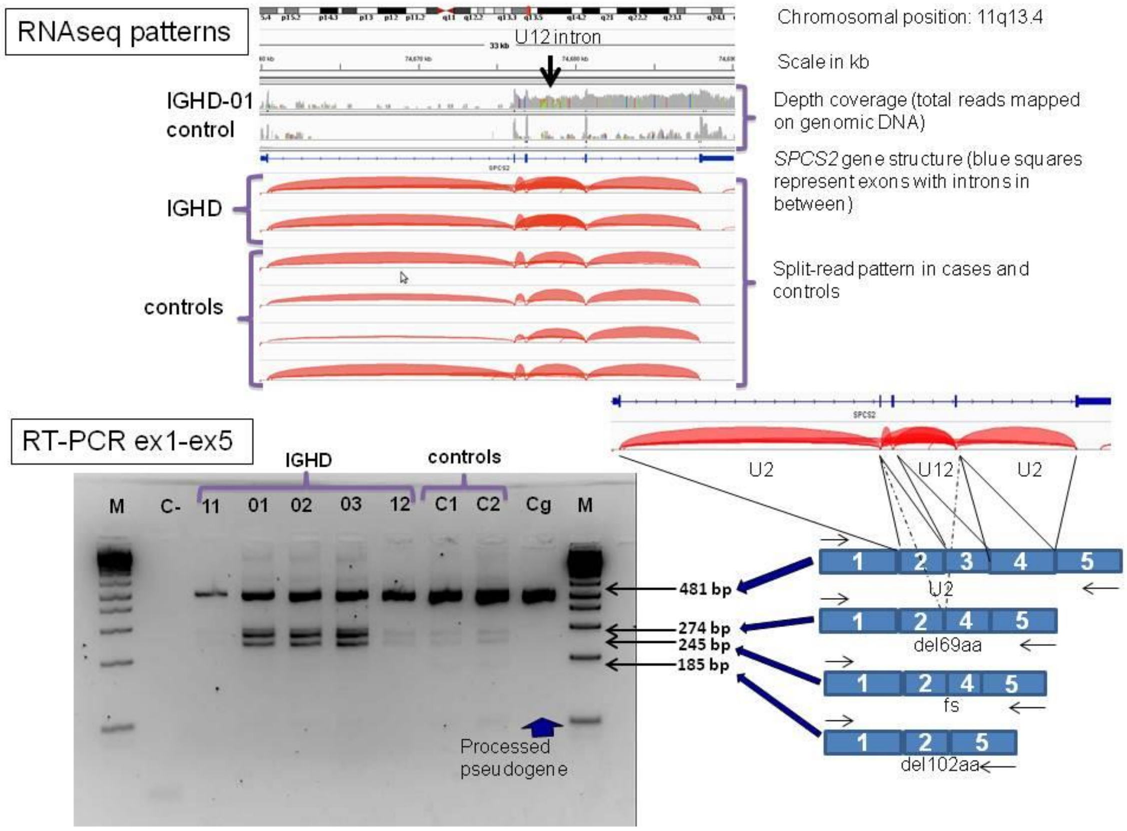

M: Molecular weight marker: 1KB Plus DNA Ladder (Invitrogen, Ref. 10787-018)

C-: Negative control (No RT or DNA product)
IGHD-11 & 12: Parents
IGHD-01, 02 & 03: Affected sisters
C1 & C2: RT-PCR from control (normal height) individuals
Cg: Amplification from genomic DNA (no RT): amplification of a processed pseudogene on chromosome.
The correspondence of every amplification product with the specific splicing processing shown on the right has been validated by sequencing (following gel purification of each product)
