# Supplementary material for: Defective minor spliceosome mRNA processing results in isolated familial growth hormone deficiency
Source: EMBO Mol Med. 2014 Jan 30;6(3):299–306. doi: 10.1002/emmm.201303573 (PMC3958305; doi:10.1002/emmm.201303573)
Supplement: Supplementary file 4 [file emmm0006-0299-sd4.pdf]

Supporting figures S1-S10: Representative examples of aberrant transcription profiles in several candidate genes with U12-processed introns in cases (IGHD 01-03) and controls by RNAseq and RT-PCR from blood RNA.
Top: RNAseq patterns visualized on the IGV tool. Under the chromosomal ideograms with the gene location and the scale in Kb, the total depth coverage (logarithmic scale) of mapped RNAseq reads in one IGHD case and one control is shown with respect to the gene (exon-intron) structure. The splicing patterns of the transcripts obtained with Bowtie2 and TopHat and provided by the IGV server for two cases and four controls are shown in red.
Bottom: RT-PCR results with a specific combination of primers, as indicated. The schemes show the exonic content of processed transcripts per amplicon with respect to the split-read pattern obtained by RNAseq. The expected consequence on the encoded protein of each aberrant transcript is shown under its scheme. The corresponding PCR products obtained in the three IGHD cases, one carrier and one or two controls are shown on the picture (ethidium bromide stained DNA amplicons after electrophoresis in agarose gels).

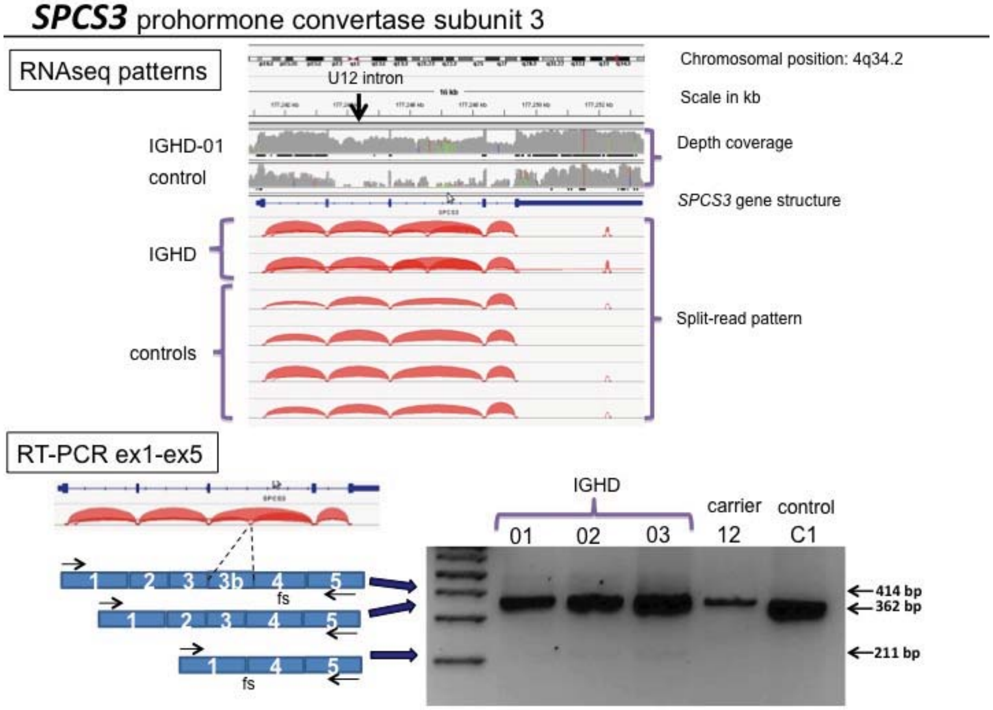

Supporting figure S1: Transcription profiles of the *SCPS3* gene. Increased intron retention (U12 and flanking U2 introns) with cryptic splicing within the downstream U2 intron is detected in cases (low proportion by RT-PCR), absent in controls. *SCPS3* also codes for a subunit of the convertase that releases signal peptide from preproghrelin.
