# Supplementary material for: Defective minor spliceosome mRNA processing results in isolated familial growth hormone deficiency
Source: EMBO Mol Med. 2014 Jan 30;6(3):299–306. doi: 10.1002/emmm.201303573 (PMC3958305; doi:10.1002/emmm.201303573)
Supplement: Supplementary file 5 [file emmm0006-0299-sd5.pdf]

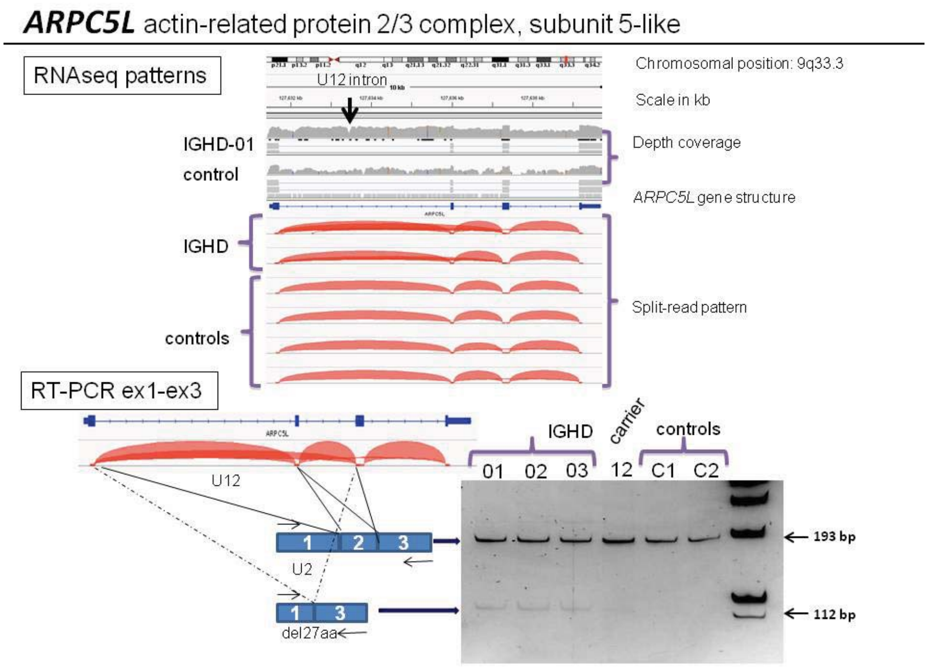

Supporting figure S2: Transcription profiles of the *ARPC5L* gene. Poor U12-type splicing with the use of alternative (cryptic) U2-type splicing and exon skipping is only seen in patients, being absent in controls. Intron retention is also higher in patients, but affects both U12 and U2-type introns.

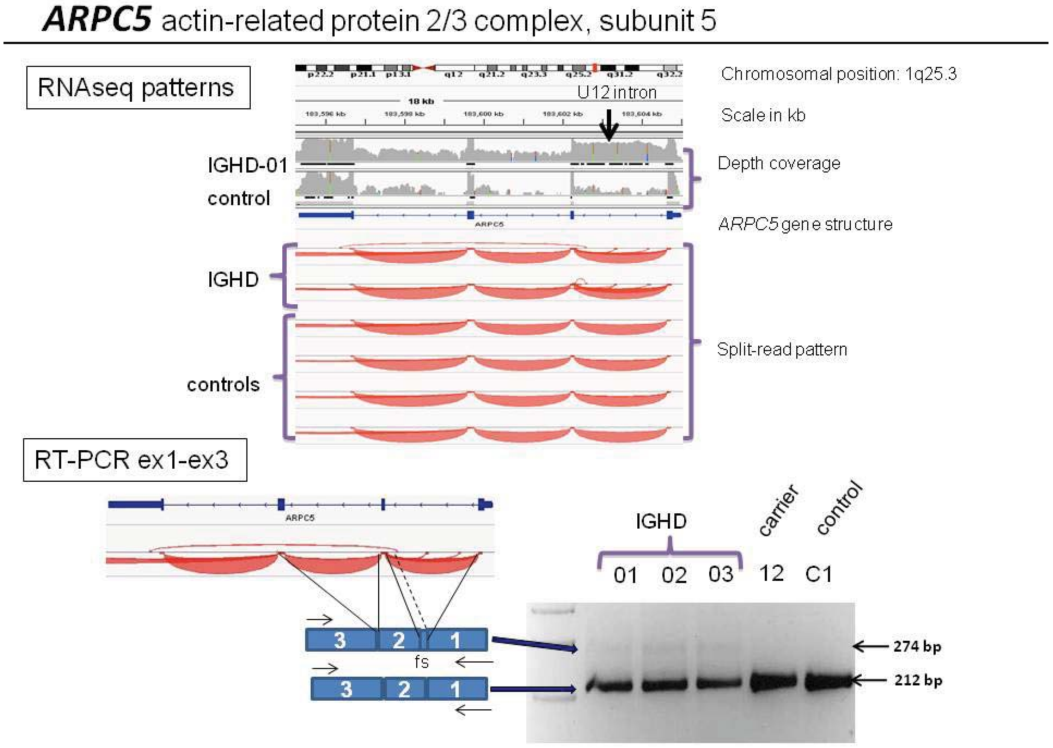

Supporting figure S3: Transcription profiles of the *ARPC5* gene. Increased U12-type intron retention is seen in patients with otherwise similar U12-type splicing. An alternative U2-type cryptic splicing within intron 1 is very weakly seen in patients, but absent in controls.
