# Supplementary material for: Defective minor spliceosome mRNA processing results in isolated familial growth hormone deficiency
Source: EMBO Mol Med. 2014 Jan 30;6(3):299–306. doi: 10.1002/emmm.201303573 (PMC3958305; doi:10.1002/emmm.201303573)
Supplement: Supplementary file 6 [file emmm0006-0299-sd6.pdf]

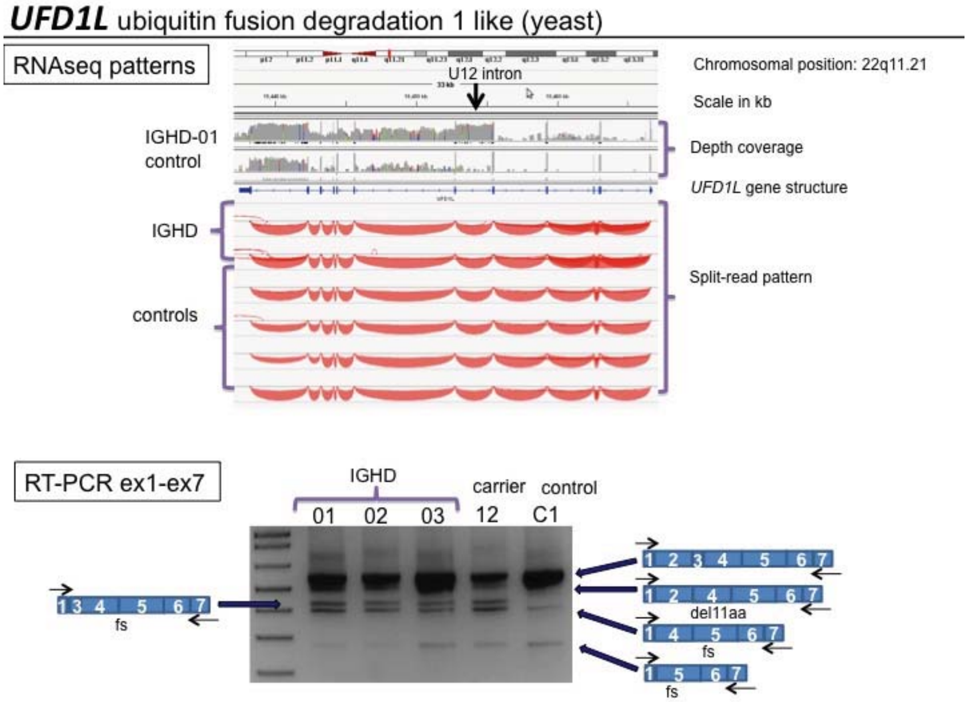

Supporting figure S4: Transcription profiles of the *UFD1L* gene. Increased intron retention (U12 and flanking U2 introns) with increased alternative splicing and exon skipping in the 5' end of the gene is seen in patients with respect to controls. Several alternative U2-type splicing are seen by RT-PCR, one of them undetected in controls.

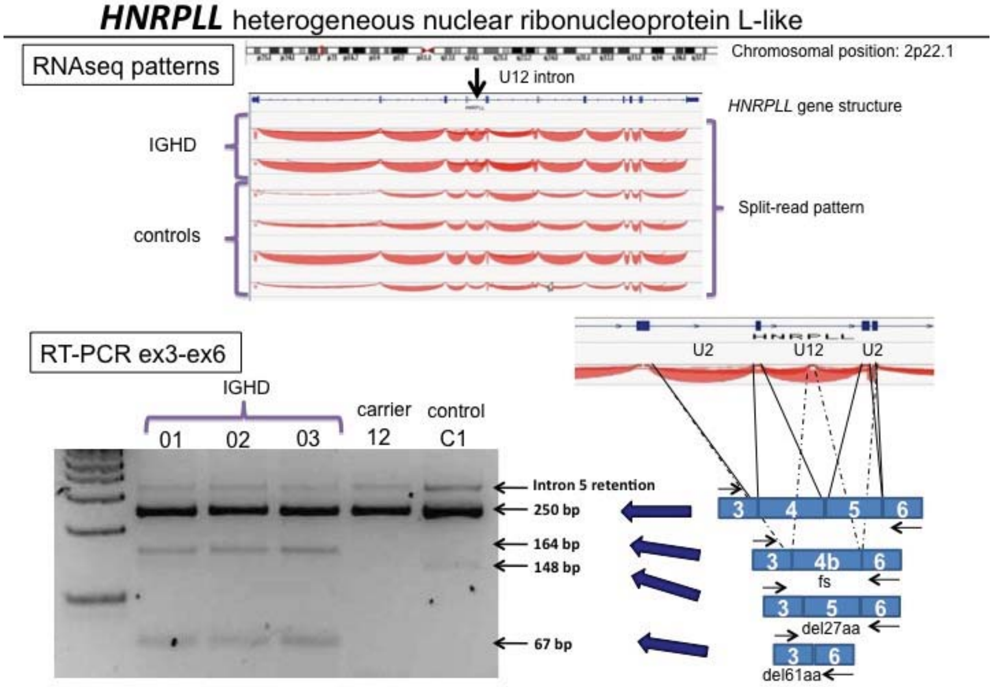

Supporting figure S5: Transcription profiles of the *HNRPLL* gene. Depth coverage is not shown due to the length of the gene. There is poorer U12-type splicing in patients with alternative (aberrant) U2-type splicing (cryptic exon and exon skipping) that is absent in controls.
