# Supplementary material for: Defective minor spliceosome mRNA processing results in isolated familial growth hormone deficiency
Source: EMBO Mol Med. 2014 Jan 30;6(3):299–306. doi: 10.1002/emmm.201303573 (PMC3958305; doi:10.1002/emmm.201303573)
Supplement: Supplementary file 7 [file emmm0006-0299-sd7.pdf]

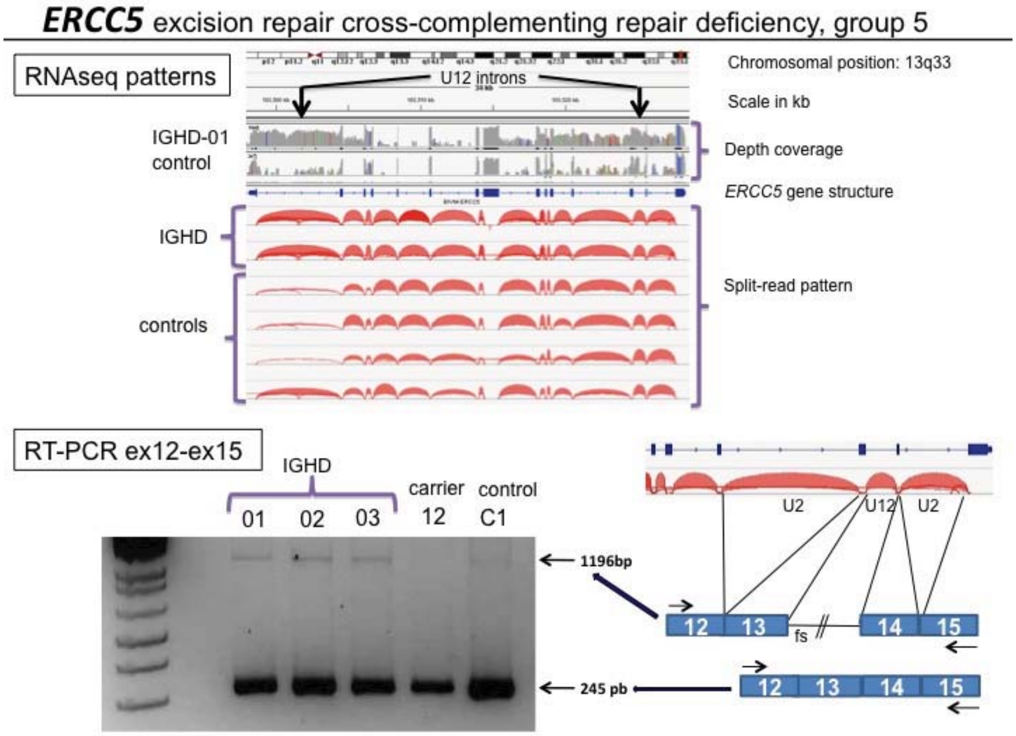

Supporting figure S6: Transcription profiles of the *ERCC5* gene. Increased U12-type intron retention is seen in patients, and also detected but weaker in controls. No alternative transcripts are detected in the studied interval.

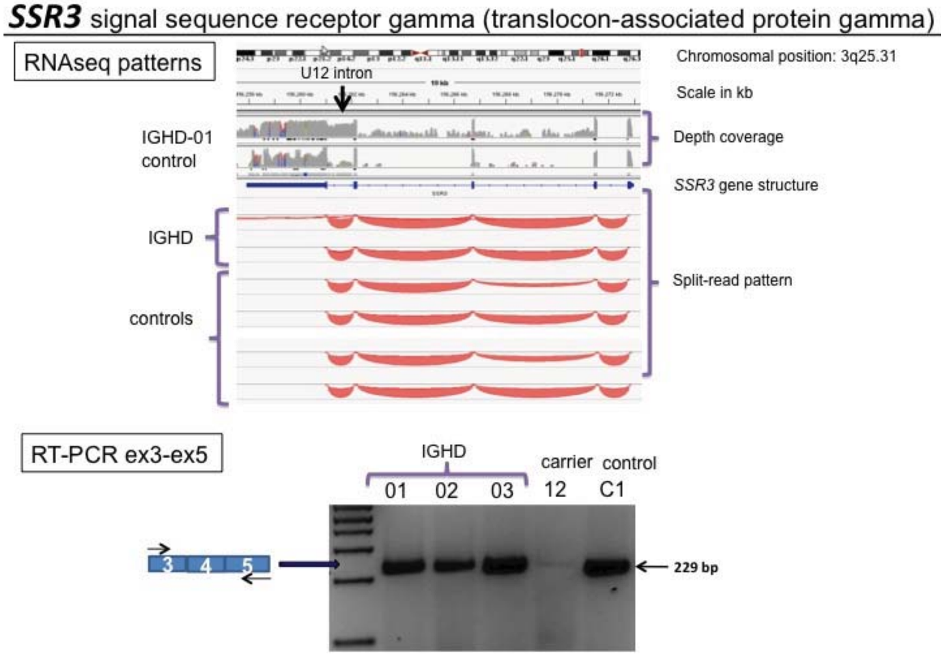

Supporting figure S7: Transcription profiles of the *SSR3* gene. Increased U12-type intron retention is seen in patients by RNAseq, although a single transcript with no differences between cases and controls is detected by RT-PCR.
