# Supplementary material for: Defective minor spliceosome mRNA processing results in isolated familial growth hormone deficiency
Source: EMBO Mol Med. 2014 Jan 30;6(3):299–306. doi: 10.1002/emmm.201303573 (PMC3958305; doi:10.1002/emmm.201303573)
Supplement: Supplementary file 8 [file emmm0006-0299-sd8.pdf]

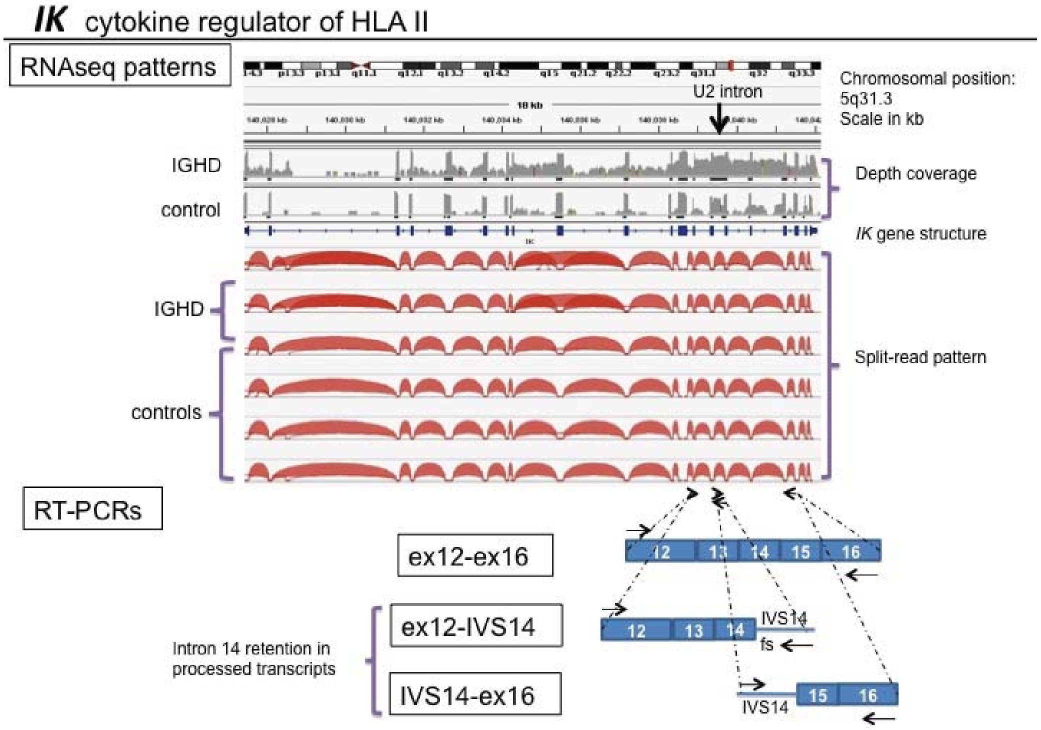


**Supporting figure S8**: Transcription profiles of the *IK* gene. There is U12-type intron retention both in cases and controls, with apparent increased alternative splicing of U2 introns upstream in cases. By RT-PCR the normally spliced and the unspliced IVS14 (pre-mRNAs) were found both in cases and controls (not shown).

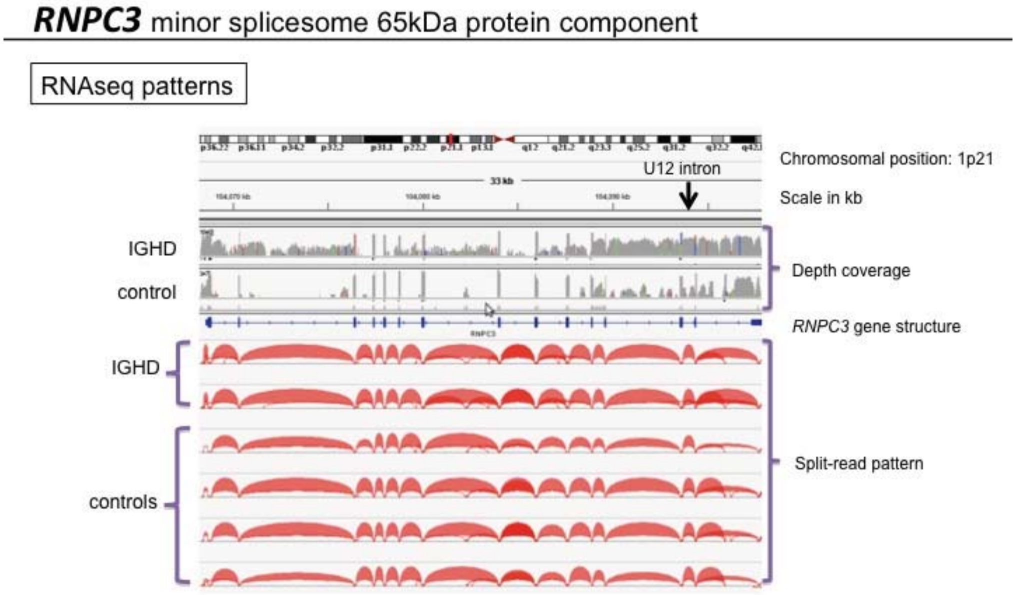


**Supporting figure S9**: Transcription profiles of the *RNPC3* gene. Increased intron retention (U12 and flanking U2 introns) is seen by RNAseq in patients, although a single transcript with no differences between

cases and controls was detected by RT-PCR. Interestingly, the *RNPC3* gene also harbors an intron processed by the minor splicesome.

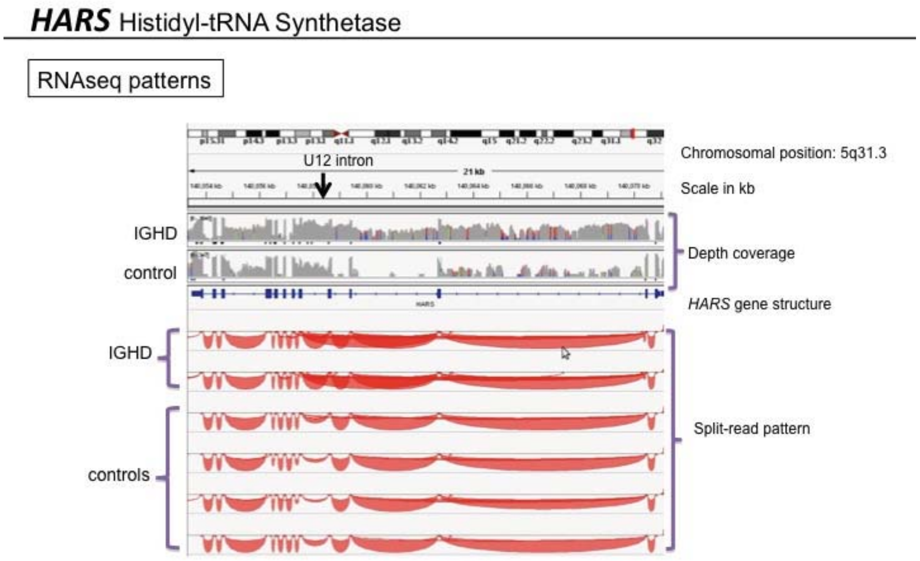


**Supporting figure S10**: RNAseq transcription profiles of the *HARS* gene. Increased intron retention (U12-type and flanking U2-type introns) along with several alternative splicing using cryptic sites is seen in patients, but also present in controls at a lower rate.
