# Supplementary material for: Defective minor spliceosome mRNA processing results in isolated familial growth hormone deficiency
Source: EMBO Mol Med. 2014 Jan 30;6(3):299–306. doi: 10.1002/emmm.201303573 (PMC3958305; doi:10.1002/emmm.201303573)
Supplement: Supplementary file 9 [file emmm0006-0299-sd9.pdf]

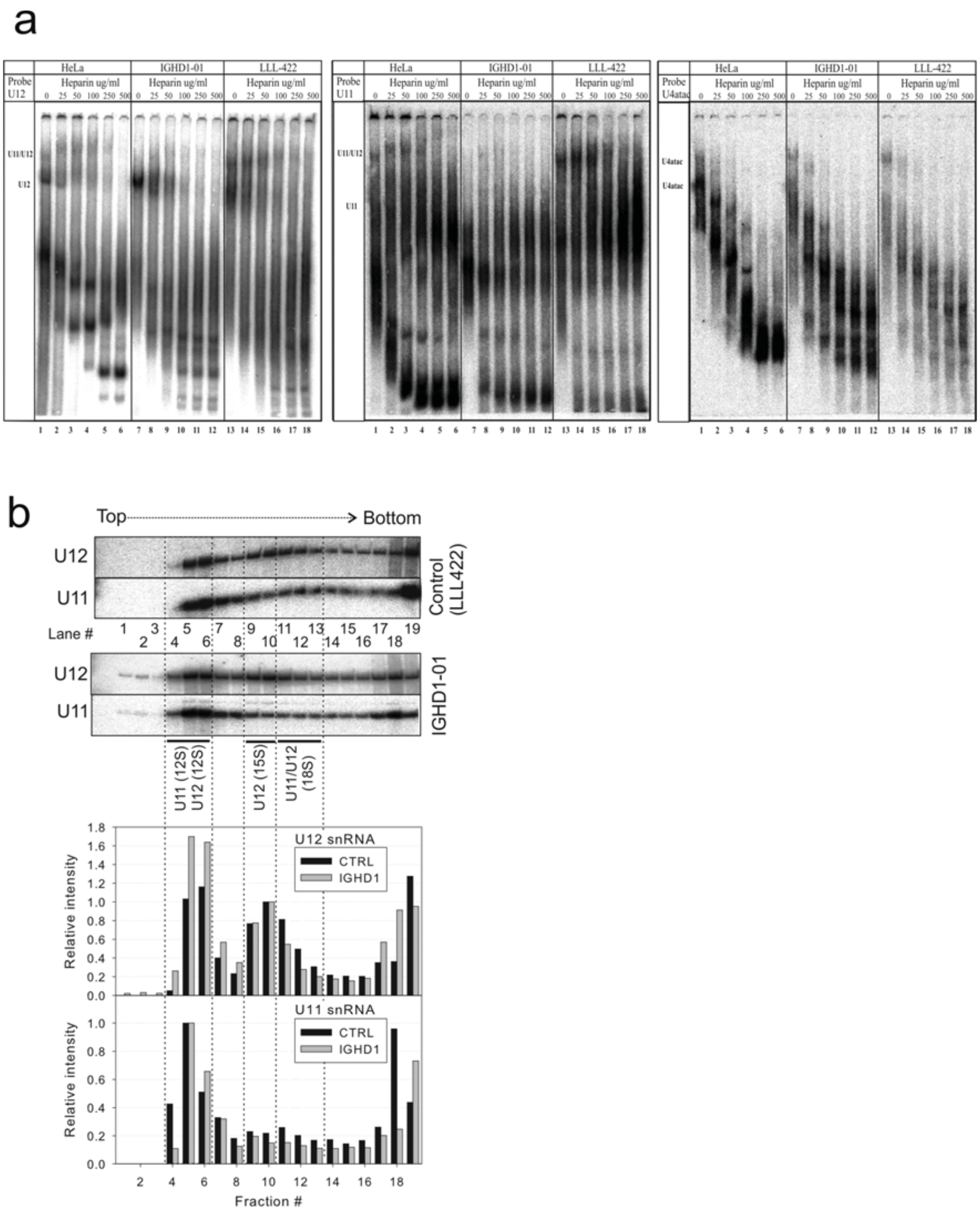

Supporting Figure S11: (a) Native gel analysis of the effect of heparin concentration on U11/U12-complex structure in nuclear extracts derived from HeLa cells and lymphoid IGHD1 patient and control cells. Increasing concentrations of heparin, as indicated in each panel, was added prior to loading followed by electrophoresis and Northern blotting and probing as indicated in each panel. (b). Glycerol gradient profile of control and patient cells and (**b**) gradient quantification (mean values of two patients and two controls). Total cell extracts were prepared and fractionated and RNA present in each fraction analyzed by Northern blot. The fraction numbers and positions of snRNP complexes are shown at the bottom. The direction of sedimentation is from top to bottom, as indicated by the arrow. The positions of U11 and U12 mono-snRNPs and the U11/U12 disnRNP are indicated in dotted line boxes.
