# Supplementary material for: Defective minor spliceosome mRNA processing results in isolated familial growth hormone deficiency
Source: EMBO Mol Med. 2014 Jan 30;6(3):299–306. doi: 10.1002/emmm.201303573 (PMC3958305; doi:10.1002/emmm.201303573)
Supplement: Supplementary file 10 [file emmm0006-0299-sd10.pdf]

Patient ID	IGHD-01		IGHD-02		IGHD-03	
Birth length in cm (SDS)	50 (0.1)		49 (-0.5)		48 (-1.1)	
	At diagnosis	1y after rGH	At diagnosis	1y after rGH	At diagnosis	1y after rGH
Chronological age (years)	15.4	16.4	8.0	9.0	6.0	7.0
Bone age (years)	12	13	6.5	7.5	3.5	5
Height in cm (SDS)	125.5 (-5.9)	137.8 (-4.2)	100.2 (-5)	114cm (-3.2)	84.5 (-6.6)	98.5 (-4.4)
Growth velocity cm/year (SDS)	----	12.8 (+31)	----	14.2 (+11.4)	----	14.6 (+9.3)
Weight for age (SDS)	-2.15	-2.33	-1.87	-1.65	-3.07	-2.56
BMI for age (SDS)	+0.8	-0.58	+0.16	-0.89	-1.5	-1.7
Total % of body fat in DXA (SDS)	44.1 (+2.9)	25.2 (-0.2)	39.2 (+2.6)	18.4 (-1.1)	36.2 (+2)	14.3 (-2.1)
OFC in cm (SDS for age)	50 (-3.1)	52.5 (-1.4)	46 (-3.9)	49.5 (-1.8)	45.5 (-3.7)	47.5 (-2.3)
OFC (SDS for height)	(-1.1)	0 (P50)	(-3.1)	(-1)	(-2.3)	(-1.7)
GH (ng/ml) → Peak after oral clonidine	<0.5		<0.5		<0.5	
IGF-I (ng/ml)	42 [237-996]	475 [130-680]	18 [52-286]	379 [67-350]	21 [45-262]	93 [52-282]
IGFBP-3 (mg/l)	0.82 [3.1-9]	5.5 [3.3-7.2]	<0.5 [1.7-7.0]	4.47 [2.2-8.0]	<0.5 [1.2-5.6]	3.65 [1.7-7.0]
Prolactin (ng/ml)	0.78 [1.6-25]	ND	2.12 [1.6-25]	ND	1.83 [1.6-25]	ND
Total ghrelin (pg/ml) (SDS)	1497 (+2.7)	887 (+0.2)	7580 (+14)	2100 (+1.8)	4875 (+8)	3223 (+4.3)
Acylated ghrelin (pg/ml) (SDS) {% of total}	28.7 (-0.6) {1.9%}	50.5 (+0.8) {5.7%}	114.3 (+1.4) {1.5%}	74 (-0.2) {3.5%}	32.3 (-1.8) {0.6%}	65.4 (-0.5) {2%}
TSH (µU/ml)	4.28 [0.36-5.5)	1.47 (0.36-5.5)	4.52 (0.36-5.5)	2.03 (0.36-5.5)	3.29 (0.36-5.5)	2.45 (0.36-5.5)
Free T4 (ng/dl)	0.91 [0.65-1.4]	0.91 [0.65-1.4	0.95 [0.65-1.4]	0.81 [0.65-1.4]	0.94 [0.65-1.4]	0.89 (0.65-1.4)
LH (mIU/ml) → Peak after GnRH	6.7 [0.2-15] → 40.1	ND	ND	ND	ND	ND
FSH (mIU/ml) → Peak after GnRH	30.3 [2-22] → 28.1	ND	[Prepubertal]	ND	[Prepubertal]	ND
Estradiol (pg/ml)	7.4 [10-400]	ND	[Prepubertal]	ND	[Prepubertal]	ND
Cortisol (µg/dl)	20.4 [5.9-22]	ND	14.3 [5.9-22]	ND	20 [5.9-22]	ND
Insulin (µIU/ml)	4.4 [4-11]	9.2	0.8 [4-11]	9.0	0.6 [4-11]	4.8

SDS: Standard Deviation Score; BMI: body mass index; DXA: dual X-ray absortiometry; OFC: occipital-frontal circumference; [normal ranges]

Supporting Table S1. Auxological and hormonal parameters of the three female probands in this family with IGHD.
