# Supplementary material for: Defective minor spliceosome mRNA processing results in isolated familial growth hormone deficiency
Source: EMBO Mol Med. 2014 Jan 30;6(3):299–306. doi: 10.1002/emmm.201303573 (PMC3958305; doi:10.1002/emmm.201303573)
Supplement: Supplementary file 11 [file emmm0006-0299-sd11.pdf]

		U12sr/U2sr		U12sr/FPKM		U12ret/U2ret		U12ret/FPKM		U12sr/U2sr	U12sr/FPKM	U12ret/U2ret	U12ret/FPKM
Intron ID	Gene	cases	controls	cases	controls	cases	controls	cases	controls	ca/co ratio	ca/co ratio	ca/co ratio	ca/co ratio
24702	*SPCS2**	0.57+/-0.1	2.12+/-0.15	3.51+/-0.37	4.83+/-0.42			0.12+/-0.01	0.02+/-0	0.27	0.73		5.45
23739	*ARPC5L**	0.27+/-0.08	0.98+/-0.2					0.62+/-0.03	0.13+/-0.01	0.28			4.63
26185	*CAPN3*	0.34+/-0.05	1.17+/-0.15					0.56+/-0.02	0.12+/-0	0.29			4.59
24373	*MTG1*	0.22+/-0.04	0.74+/-0.14					0.54+/-0	0.12+/-0.02	0.30			4.50
24535	*C11orf10*	0.48+/-0.04	1.11+/-0.11	4.99+/-0.51	6.06+/-0.31			0.3+/-0.01	0.13+/-0	0.44	0.82		2.28
24252	*SMC3*	0.48+/-0.04	0.93+/-0.12			0.13+/-0.01	0.06+/-0.01	0.31+/-0.02	0.07+/-0.01	0.51		2.06	4.44
29591	*MAEA*	0.41+/-0.03	0.65+/-0.05					0.32+/-0.01	0.1+/-0.01	0.64			3.27
30117	*HARS*			0.29+/-0.04	1.52+/-0.14			0.39+/-0.04	0.04+/-0		0.19		9.31
23312	*TM2D1*			1.62+/-0.23	7.03+/-0.72			0.2+/-0.01	0.17+/-0.01		0.23		1.16
24426	*PSMA1*			0.53+/-0.02	1.88+/-0.36	0.17+/-0.01	0.07+/-0.01	0.47+/-0.05	0.11+/-0		0.28	2.36	4.43
31089	*DOCK5*			1.26+/-0.18	4.4+/-0.36	19.28+/-3.28	1.58+/-0.19	1.09+/-0.06	0.05+/-0.01		0.29	12.17	23.26
26813	*DERL2*			0.98+/-0.18	3.12+/-0.24			0.41+/-0.05	0.14+/-0.01		0.31		2.93
30306	*SRPK2*			1.87+/-0.3	4.63+/-0.24			0.17+/-0	0.01+/-0		0.40		19.02
24264	*BRWD2*			3.53+/-0.06	6.6+/-0.03			0.42+/-0.06	0.16+/-0.01		0.54		2.58
25647	*RABGGTA*			1.53+/-0.05	2.72+/-0.07			0.97+/-0.01	0.39+/-0.02		0.56		2.47
30790	*RINT1*			3.85+/-0.56	6.6+/-1.15			0.16+/-0.01	0.08+/-0.01		0.58		2.10
30523	*PPIL4*			5.66+/-0.22	8.13+/-0.52			0.48+/-0.01	0.11+/-0.01		0.70		4.58
25130	*VEZT*			2.3+/-0.05	3.15+/-0.35			0.03+/-0	0.01+/-0		0.73		2.77
28920	*RAF1*			0.6+/-0.02	0.8+/-0.02	0.11+/-0.01	0.04+/-0.01	0.82+/-0.1	0.11+/-0.02		0.75	3.02	7.14
26790	*TRAPPC2L*			5.04+/-0.6	6.41+/-0.43			0.73+/-0.08	0.17+/-0		0.79		4.30
25244	*SDS3*			4.77+/-0.95	5.87+/-0.29	0.3+/-0.02	0.07+/-0.01	1.05+/-0.1	0.09+/-0.01		0.81	4.36	11.50
28728	*UFD1L**			4.08+/-0.75	4.48+/-0.08	1.96+/-0.12	0.42+/-0.06	0.35+/-0.03	0.03+/-0		0.91	4.67	12.32

Supporting Table S2. List of U12-type introns in genes with significantly defective splicing on RNAseq data from mononuclear blood cells of IGHD cases. Only genes showing consistent and significantly altered values (> or <2SDS in cases with respect to controls) in two or more of the four calculations are shown, ranked by the U12-type splicing inefficiency measured by the U12sr/U2sr ratio.

Intron ID: intron identifier in U12DB

U12sr: Spliced reads across two exons joined by U12-type processing

U2sr: Spliced reads across two exons joined by U2-type processing

U12ret: Reads within a non-spliced U12-type intron

U2ret: Reads within a non-spliced U2-type intron

FPKM: Fragments Per Kilobase of transcript per Million fragments mapped

ca/co ratio: ratio of the specific values in cases versus controls

*Genes with significant expression and/or function during pituitary development. *UFD1L* encodes a developmentally expressed ubiquitination protein, deleted in CATCH 22 syndrome, expressed in developing anterior pituitary.
