# Supplementary material for: Defective minor spliceosome mRNA processing results in isolated familial growth hormone deficiency
Source: EMBO Mol Med. 2014 Jan 30;6(3):299–306. doi: 10.1002/emmm.201303573 (PMC3958305; doi:10.1002/emmm.201303573)
Supplement: Supplementary file 12 [file emmm0006-0299-sd12.pdf]

Gene	U12-type intron	RT-PCR	Oligo Forward (5'-3')	Oligo Reverse (5'-3')	Tan (°C)	cDNA product(s) (bp)	cDNA (aberrant) (bp)	Result in patients' blood RNA	Result in controls' blood RNA	gDNA
ARPC5	1	exons 1-3	GAAGGTGGACGTGGATGAAT	GCACCTTCAAGACAATGCTG	59	212	274	normal & aberrant (weak) U12 processing (cryptic)	normal expression and processing	No product
ARPC5L	1	exons 1-3	AATTTGTGGACGAGCAGGAG	GTGAGCACTTTCAGCACCAC	59	193	112	normal & aberrant U12 processing (cryptic)	normal expression and processing	No product
ERCC5	1 & 13	exons 12-15	GGCCTGGAACCTCTCCTAAA	GGAATCAATTCGGAGCTGTG	59	333	1196	normal & aberrant U12 processing (intron retention)	normal expression and processing (lower proportion)	No product
HNRPLL	4	exons 3-6	AATCCGCTTTATCCAATTACAG	CCCAACTGTCATTGTCATTCC	59	250 & 148	164 & 67	normal & aberrant U12 processing (cryptic exon)	normal expression and processing	No product
IK	14	exons 12-16	GGACGTTGACAAAGGACCTG	CCCTTCTTGTTACCCTGGTC	58	246	none	normal expression and processing	normal expression and processing	No product
IK	14	exon 12-intron 14	GGACGTTGACAAAGGACCTG	GTGCTCTGGGAAGGAACAAG	58	none	249	U12 intron retention detected in cDNA	U12 intron retention detected in cDNA	No product
IK	14	intron 14-exon 16	TTCATGTTGGGGCTTCTAGC	CCCTTCTTGTTACCCTGGTC	58	none	248	U12 intron retention detected in cDNA	U12 intron retention detected in cDNA	No product
MAPK8	6	exons 5-8	GCATGGGCTACAAGGAAAAC	ACAAATCCCTTGCCTGACTG	60	297	216	normal & aberrant U12 processing (cryptic)	normal & aberrant U12 processing (similar)	No product
NUP210	7	exons 6-9	GAAGATCAGGCAAGGGAAAA	TCACCAGGGTGAACAGTGAA	59	276	none	normal expression and processing	normal expression and processing	No product
NUP210L	7	exons 6-9	TGGTTCAAGGGAGAGTGACA	CCCACCTCTAGACTCCATCG	59	292	none	No signal + no reads from RNAseq	No signal + no reads from RNAseq	No product
PSCK1	8	exons 7-10	TAGCCCAGAAGGCTTTTGAA	GAGGTCCAGACAACCAGGTG	59	397 & 184	none	No signal + no reads from RNAseq	No signal + no reads from RNAseq	No product
RNPC3	13	exons 12-14	ATCAGAAACACAGCGGATCA	TTCCTTCCTTAGGATCTTGTTTTG	59	200	none	normal expression and processing	normal expression and processing	No product
SLC12A7	10	exons 9-12	TCTACTTCCCTTCCGTGACC	CATGCCGATGACCAGGTT	60	218	none	normal expression and processing	normal expression and processing	Product
SPCS2	3	exons 1-5	CGTAGCGGCTTGTTGGATAA	ACTTTGTGAACTCGGCTTCC	61	481	274, 245 & 185	normal & aberrant (high proportion) U12 processing	normal & aberrant (low proportion) U12 processing	481 (pseudogene)
SPCS3	2	exons 1-5	ACCGCCTTCAAAGACAGGAG	ACGTTCCAAGACAGGGTCAA	62	362	414 & 211	normal & aberrant (low proportion) processing	normal expression and processing	No product
SSR3	4	exons 3-5	GATTTCAGCCGCAATCTCTC	CATCCTCCCTCTTCTGTGCT	56	229	none	normal expression and processing	normal expression and processing	No product
UFD1L	5	exons 1-7	GTTGCATTGCCTGAGAGGAG	CCATCACACGCAGTTCGTAG	60	536, 503, 403 & 248	430	normal expression with alternative transcripts	normal & weak aberrant processing	No product
UFD1L	5	exons 5-7	GCCACCTACTCCAAATTCCAA	CCATCACACGCAGTTCGTAG	59	160	none	normal expression and processing	normal expression and processing	No product

Supporting Table S3. List of genes with U12-type introns analyzed by RT-PCR, including primers, PCR conditions and final results
